# The laminin–keratin link shields the nucleus from mechanical deformation and signalling

**DOI:** 10.1038/s41563-023-01657-3

**Published:** 2023-09-14

**Authors:** Zanetta Kechagia, Pablo Sáez, Manuel Gómez-González, Brenda Canales, Srivatsava Viswanadha, Martín Zamarbide, Ion Andreu, Thijs Koorman, Amy E. M. Beedle, Alberto Elosegui-Artola, Patrick W. B. Derksen, Xavier Trepat, Marino Arroyo, Pere Roca-Cusachs

**Affiliations:** 1 Institute for Bioengineering of Catalonia (IBEC), Barcelona Institute of Science and Technology (BIST), Barcelona, Spain; 2 Laboratori de Càlcul Numèric (LàCaN), Universitat Politècnica de Catalunya, Barcelona, Spain; 3 Institut de Matemátiques de la UPC–BarcelonaTech (IMTech), Barcelona, Spain; 4 Cell and Tissue Mechanobiology Laboratory, The Francis Crick Institute, London, UK; 5 Department of Physics, King’s College London, London, UK; 6 University of Barcelona, Barcelona, Spain; 7 Instituto Biofisika (UPV/EHU, CSIC), University of the Basque Country, Leioa, Spain; 8 Ikerbasque, Basque Foundation for Science, Bilbao, Spain; 9 Department of Pathology, University Medical Center Utrecht, Utrecht, The Netherlands; 10 Centro de Investigación Biomédica en Red en Bioingeniería, Biomateriales y Nanomedicina (CIBER-BBN), Barcelona, Spain; 11 Institució Catalana de Recerca i Estudis Avançats (ICREA), Barcelona, Spain; 12 Centre Internacional de Mètodes Numèrics en Enginyeria (CIMNE), Barcelona, Spain

**Keywords:** Intermediate filaments, Mechanotransduction, Coarse-grained models, Integrins, Rheology

## Abstract

The mechanical properties of the extracellular matrix dictate tissue behaviour. In epithelial tissues, laminin is a very abundant extracellular matrix component and a key supporting element. Here we show that laminin hinders the mechanoresponses of breast epithelial cells by shielding the nucleus from mechanical deformation. Coating substrates with laminin-111—unlike fibronectin or collagen I—impairs cell response to substrate rigidity and YAP nuclear localization. Blocking the laminin-specific integrin β4 increases nuclear YAP ratios in a rigidity-dependent manner without affecting the cell forces or focal adhesions. By combining mechanical perturbations and mathematical modelling, we show that β4 integrins establish a mechanical linkage between the substrate and keratin cytoskeleton, which stiffens the network and shields the nucleus from actomyosin-mediated mechanical deformation. In turn, this affects the nuclear YAP mechanoresponses, chromatin methylation and cell invasion in three dimensions. Our results demonstrate a mechanism by which tissues can regulate their sensitivity to mechanical signals.

## Abstract (web summary)

Laminin, an important component of the extracellular matrix supporting the epithelium, hinders the typical mechanoresponse of epithelial cells to an increase in substrate stiffness, by protecting the cell nucleus from mechanical deformation.

## Main

Extracellular matrix (ECM) parameters, such as its composition or mechanical properties, shape cellular responses and altered cell–ECM interactions drive pathological conditions such as cancer and fibrosis^1^. Different combinations of ECM molecule trigger intracellular events that propagate cell- and tissue-specific responses through changes in gene expression and signalling. This signalling is mediated by ECM interactions with their receptors on the cell membrane called integrins^1,2^. Integrins also respond to changes in the ECM mechanical properties^2^ in a process known as mechanotransduction. Increased ECM stiffness can lead to the activation, clustering and maturation of integrin adhesions. In turn, this triggers cytoskeletal rearrangements and a buildup of intracellular tension that can propagate to the cell nucleus, where it regulates the nuclear localization and activity of transcriptional regulators such as YAP^2,3^.

This framework of integrin-mediated cell response to increased ECM stiffness and mechanotransduction to the nucleus has largely been studied for ECM components such as fibronectin and collagen^2–4^, as well as for reconstituted basement membranes (BMs) such as Matrigel^5–7^. However, the specific role of laminin, a common substrate for all epithelial tissues, is unclear. Laminin forms an important part of the BM underlying the epithelial tissues and guides pivotal cellular processes ranging from healthy epithelial homoeostasis to cancer metastasis^8–10^. Changes in BM composition or mechanical properties are critical in several stages of cancer progression, regulating both tissue organization and tumour invasiveness^9,10^. This role of the BM has been particularly well characterized and reported for the case of breast cancer^5,11,12^. We, thus, sought to investigate how cells respond to increased tissue rigidity on a laminin-based extracellular environment, and how these changes can influence mechanotransduction in mammary epithelial cell models.

## Laminin-111 hinders cell response to rigidity

**Fig. 1:**
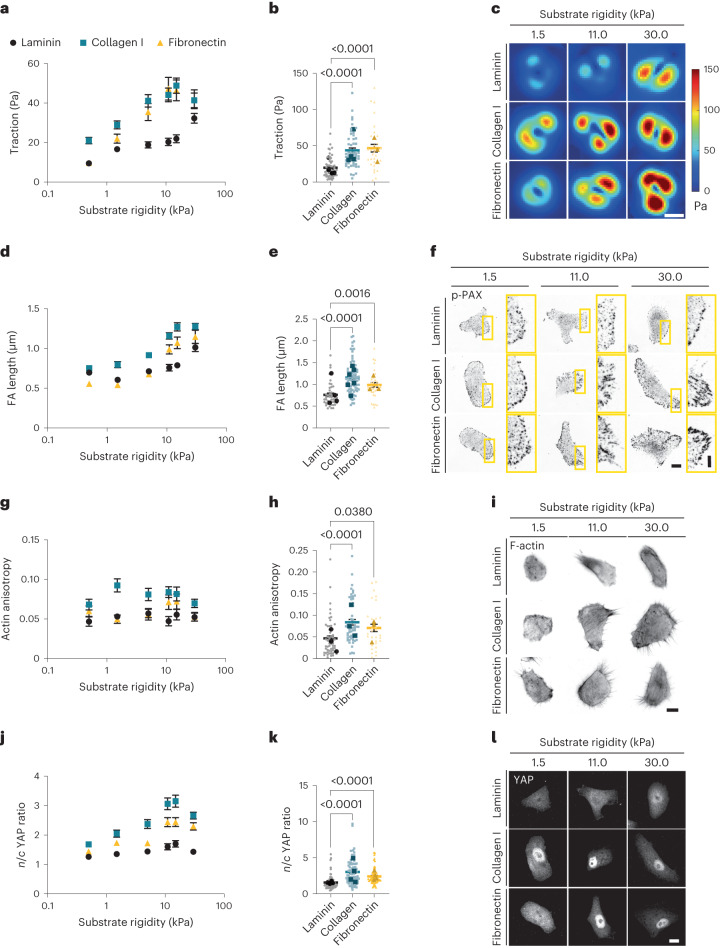
Laminin coating impairs cell mechanosensing in response to substrate rigidity. **a**, Average values of cell tractions on PAA gels of different rigidities (0.5–30.0 kPa) and substrate coatings (*n* = 56/66/41, 59/93/45, 80/66/26, 55/50/30, 51/45/32 and 79/64/23 cells for laminin/collagen I/fibronectin substrates and increasing rigidity; mean of at least three independent experiments). The effect of both rigidity and substrate coating is significant (*P* < 0.0001, two-way analysis of variance (ANOVA)). **b**, Cell tractions at 11 kPa (*n* = 55/50/30 cells for laminin/collagen I/fibronectin, where bigger and darker points represent the averages of individual experiments; *P* < 0.0001, one-way ANOVA, Tukey’s multiple comparisons test). **c**, Corresponding example colour maps of traction forces for different rigidities and substrate coatings. Scale bar, 10 μm. **d**, Average values of FA length from p-Pax stainings (*n* = 69/91/48, 47/75/35, 63/89/37, 43/80/31, 53/64/52 and 58/110/48 cells for laminin/collagen I/fibronectin substrates and increasing rigidity; mean of at least three independent experiments). The effect of both rigidity and substrate coating is significant (*P* < 0.0001, two-way ANOVA). **e**, FA length at 11 kPa (*n* = 43/80/31 cells for laminin/collagen I/fibronectin; *P* < 0.0001, one-way ANOVA, Tukey’s multiple comparisons test). **f**, Corresponding example images of p-Pax stainings. Scale bar, 10 μm. The image on the right-hand side of each pair corresponds to the yellow rectangle in the image on the left-hand side. Scale bar, 4 μm. **g**, Quantification of actin anisotropy (*n* = 43/53/51, 51/52/43, 47/62/36, 54/54/30, 44/48/33 and 76/50/29 cells for laminin/collagen I/fibronectin substrates and increasing rigidity; mean of at least three independent experiments). The effect of substrate coating is significant (*P* < 0.0001, two-way ANOVA). **h**, Actin anisotropy at 11 kPa (*n* = 54/54/30 cells for laminin/collagen I/fibronectin; *P* < 0.0001, one-way ANOVA, Tukey’s multiple comparisons test). **i**, Corresponding images of phalloidin stainings. Scale bar, 10 μm. **j**, Quantification of *n*/*c* YAP ratio (*n* = 108/95/119, 60/68/80, 68/61/70, 78/75/57, 59/62/72 and 123/111/84 cells for laminin/collagen I/fibronectin substrates and increasing rigidity; mean of at least three independent experiments). The effect of both rigidity and substrate coating is significant (*P* < 0.0001, two-way ANOVA). **k**, *n*/*c* YAP ratio at 11 kPa (*n* = 78/75/57 cells for laminin/collagen I/fibronectin; *P* < 0.0001, one-way ANOVA, Tukey’s multiple comparisons test). **l**, Corresponding example images of YAP stainings. Scale bar, 10 μm. The error bars represent mean ± standard error of the mean (s.e.m.).

To study the role of laminin in cell mechanoresponses, we focused on the well-known breast epithelial model of MCF10A cells and one of the main types of laminin present in breast epithelia in vivo and in vitro models, namely, laminin-111 (refs. ^12–14^). Mechanoresponses such as the nuclear localization of YAP are abrogated by cell–cell contact and cadherin ligation, and high nuclear YAP levels are associated with E-cadherin-deficient breast tumours^15,16^. To isolate the role of cell–ECM interactions from those mediated by cell–cell contact, we studied single cells. We first compared the mechanoresponses of MCF10A cells on laminin-111 (referred to as laminin hereafter) with those on collagen I and fibronectin. To this end, we used polyacrylamide (PAA) gels of rigidities between 0.5 and 30.0 kPa, thereby encompassing the range of soft (healthy) and stiff (malignant) breast tissue^17^. We coated gels with laminin, collagen I or fibronectin, and first quantified the cell tractions on each condition through traction force microscopy^18^. We found that cells exerted much lower tractions when seeded on laminin than collagen I or fibronectin substrates (Fig. 1a–c).

**Extended Data Fig. 1:**
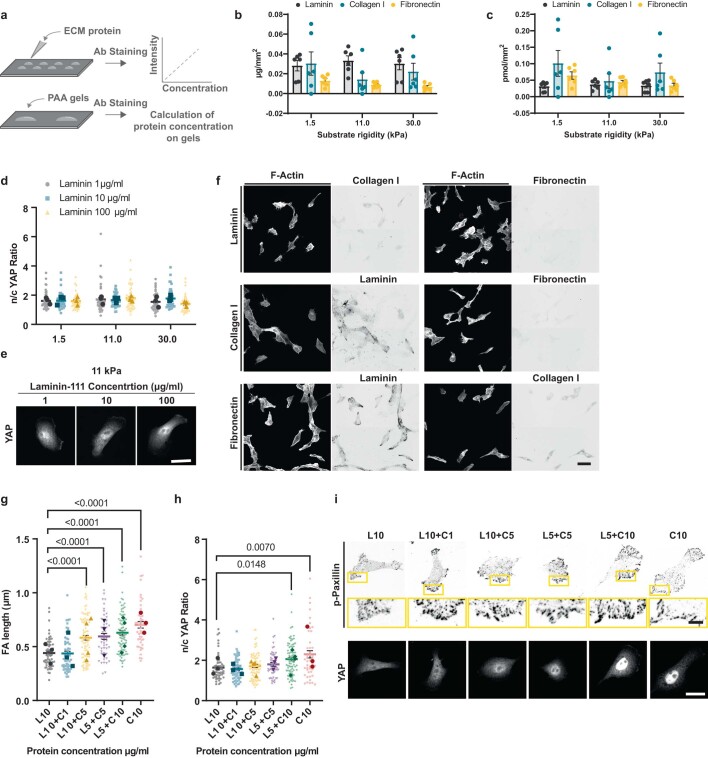
Characterization of PAA gel coating efficiency and the effect of coating composition in cell mechanoresponses. **a** Experimental design for protein coating concentration measurements. Known amounts of proteins (laminin, collagen I, and fibronectin) adhered on hydrophobic surfaces were stained, and a standard curve was generated correlating fluorescent intensity and the amount of protein. At the same time, PAA gels of 1.5, 11 and 30 kPa were coated with laminin, collagen I, and fibronectin and stained for the corresponding protein. The average amount of protein per mm^2^ was calculated according to the intensity of the signal of the gels. **b** Quantification of the μg/mm^2^ of PAA gel protein coating for 1.5, 11 and 30 kPa gels (*P* > 0.05 two-way ANOVA, n = 6 gels from 3 independent experiments). **c** Quantification of the pmol/mm^2^ of PAA gel protein coating for 1.5, 11 and 30 kPa gels (*P* > 0.05 two-way ANOVA, n = 6 gels from 3 independent experiments). **d** n/c YAP ratios of MCF10A cells seeded on 1.5, 11 and 30 kPa PAA gels coated with 1, 10 or 100 μg/ml of Laminin (n = 44/43/42, 41/40/40, 33/42/41 cells on laminin 1/10/100 μg/ml and substrates of increasing rigidity; mean of 3 independent experiments). Neither the effects of stiffness (*P* = 0.1742) nor coating density (*P* = 0.5774) was significant (two-way ANOVA, Turkey’s multiple comparison test). **e** Corresponding YAP stainings of MCF10A cells seeded on 11 kPa PAA gels coated with 1, 10 or 100 μg/ml of Laminin; scale bar is 20 μm. **f** Stainings of MCF10A cells seeded on 11 kPa PAA gels coated with laminin, collagen I or fibronectin; scale bar is 10 μm. **g** FA length of cells seeded on 11 kPa PAA gels coated with 10 μg/ml Laminin (L10) or collagen I (C10) substrates or a combination of the two. L indicates laminin and C collagen I. The number corresponds to the μg/ml used for coating (one-way ANOVA, Dunnetts’s multiple comparison test, n = 59/58/81/48/80/46 cells on L10/L10+C1/ L10+C5/ L5+C5/ L5+C10/ C10 coated gels; mean of at least 3 independent experiments). **h** n/c YAP ratio of cells seeded on 11 kPa PAA gels coated with pure Laminin (L10) or collagen I (C10) substrates or a combination of the two. L indicates laminin and C collagen I. The number corresponds to the μg/ml used for coating. (one-way ANOVA, Dunnetts’s multiple comparison test, n = 54/56/79/49/78/44 cells on L10/L10+C1/ L10+C5/ L5+C5/ L5+C10/ C10 coated gels; mean of at least 3 independent experiments). **i** Example images of p-Pax and YAP stainings of cells seeded on 11 kPa PAA gels coated with different laminin and collagen I ratios; scale bar is 20 μm (main images)/5 μm (zoomed images). Error bars represent mean ± s.e.m.

Increased rigidity induces intracellular mechanoresponses, typically including the growth of focal adhesion (FA), formation of actin stress fibres and YAP nuclear translocation^2^. In agreement with their lower traction forces, cells seeded on laminin-coated PAA gels exhibited short FA length (as quantified through phosphorylated paxillin (p-Pax) stainings), low actin fibre alignment (as quantified by fibre anisotropy) and low levels of YAP nuclear localization, with average nuclear-to-cytoplasmic (*n*/*c*) ratios below 2 (Fig. 1d–l). In contrast, all these responses were higher on collagen I and fibronectin substrates and markedly increased with rigidity (Fig. 1d–l). Through different controls, we checked that the differential response of laminin was not due to differences in ECM coating densities, ECM deposition or a specific cell type (Supplementary Note 1 and Extended Data Fig. 1). Taken together, these results indicate that laminin hinders cell responses to rigidity.

## Integrin α6β4 impedes the nuclear localization of YAP

**Fig. 2:**
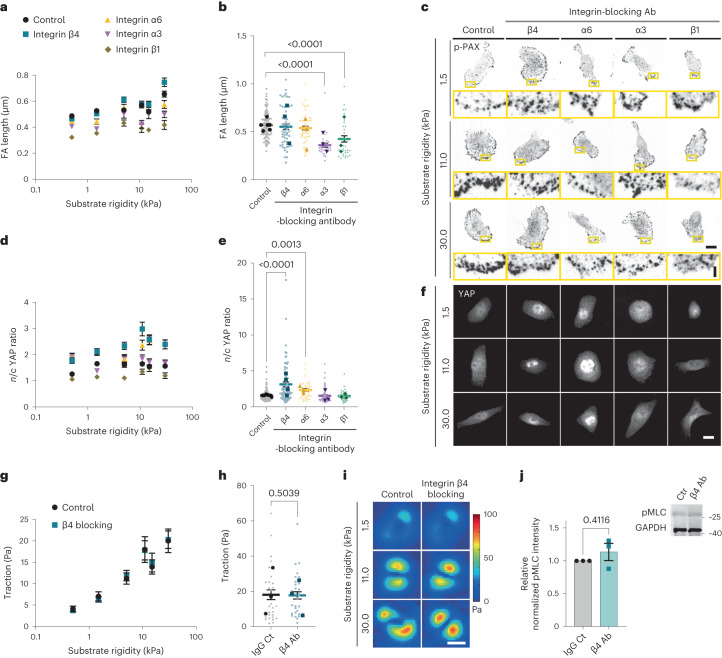
Integrin α6β4 alters nuclear mechanosensing on laminin. **a**, Average values of the FA length of MCF10A cells seeded on laminin-coated PAA gels of different rigidities on treatment with different integrin-blocking antibodies (*n* = 83/59/39/38/31, 94/46/46/41/37, 92/52/45/29/29, 99/53/46/24/32, 48/32/21/32/35 and 62/35/21/36/29 cells treated with control/β4/α6/α3/β1 antibodies for substrates of increasing rigidity; mean of at least three independent experiments). The effect of both integrin blocking and substrate stiffness is significant (*P* < 0.0001, two-way ANOVA). **b**, FA length at 11 kPa (99/53/46/24/32 cells treated with control/β4/α6/α3/β1 antibodies; *P* < 0.0001, Kruskal–Wallis test, Dunn’s multiple comparison test). **c**, Sample p-Pax stainings of MCF10A cells treated with control or β4-/α6-/α3-/β1-blocking antibodies for 1.5, 11.0 and 30.0 kPa substrate stiffness. Scale bars, 10 μm (main images)/2 μm (zoomed images). **d**, Average values of *n*/*c* YAP ratio of MCF10A cells seeded on laminin-coated PAA gels of different rigidities on treatment with different integrin-blocking antibodies (*n* = 90/61/28/30/33, 104/72/38/58/26, 101/73/49/43/35, 121/70/40/69/38, 94/51/39/51/32, 105/62/51/46/34 of control or β4-/α6-/α3-/β1-antibodies-treated cells for substrates of increasing rigidity; mean of at least three independent experiments). The effect of both integrin blocking and substrate stiffness is significant (*P* < 0.0001, two-way ANOVA). **e**, *n*/*c* YAP ratios at 11 kPa (121/70/40/69/38 cells treated with control or β4/α6/α3/β1 antibodies; *P* < 0.0001, Kruskal–Wallis test, Dunn’s multiple comparisons test). The error bars represent mean ± s.e.m. **f**, Sample YAP stainings of MCF10A cells treated with control or β4-/α6-/α3-/β1-blocking antibodies for 1.5, 11.0 and 30.0 kPa substrate stiffness. Scale bar, 10 μm. **g**, Average values of MCF10A cell tractions seeded on laminin-coated PAA gels of different rigidities and treated with control or β4-blocking antibodies (*n* = 27/29, 27/30, 39/36, 28/30, 32/32 and 28/29 of control or β4-antibody-treated cells for substrates of increasing rigidity; mean of three independent experiments). The effect of rigidity is significant (*P* < 0.0001, two-way ANOVA). **h**, Cell tractions at 11 kPa (*n* = 28/30 control or β4-antibody-treated cells; two-tailed unpaired *t*-test). **i**, Corresponding colour maps of cell traction forces. Scale bar, 10 μm. **j**, Western blot and quantification for phospho-MLC2 (pMLC) levels in MCF10A cells treated with control (Ctr) or integrin-β4-blocking antibody (β4 Ab) on normalizing to the control-treated cells (*n* = 3 independent experiments, two-tailed paired *t*-test). The error bars represent mean ± s.e.m. Source data

Adhesion to laminin is mainly mediated by α6β4, α7β1, α6β1 and α3β1 integrin dimers^19,20^. To determine their involvement, we blocked integrin β4, α6, α3 and β1, using blocking antibodies. In all the cases, FAs remained small (Fig. 2a–c). However, blocking either integrin β4 or its binding partner integrin α6 increased YAP *n*/*c* ratios in a rigidity-dependent way (Fig. 2d–f), to the levels found on collagen I or fibronectin substrates (Fig. 1j). The same trend was observed on integrin β4 depletion with siRNA (Extended Data Fig. 3a–c). Interestingly, blocking α6β4 integrins in keratinocytes has been previously reported to increase myosin phosphorylation and cell contractility, also leading to increased YAP *n*/*c* ratios^21^. However, in our case, the effect of integrin α6β4 blocking was specific to nuclear YAP levels, without affecting FAs (Fig. 2a–c), traction forces (Fig. 2g–i) or myosin light chain phosphorylation (pMLC) (Fig. 2j). Confirming the prominent role of nuclear YAP mechanosensing in conditions dominated by cell–matrix rather than cell–cell contacts^16,22^, blocking β4 integrins had a similar effect at the edge of cell colonies, but did not affect cells in the centre of confluent colonies, where *n*/*c* YAP ratios remained close to 1 (Extended Data Fig. 3d–f). Further supporting the mechanosensitive role of integrin β4, we found that it exhibited a rigidity-dependent increase in expression (Extended Data Fig. 3g), which was absent when cells were seeded on collagen-I-coated substrates, suggesting that this is a laminin-specific response.

Similar to MCF10A cells, blocking integrin β4 and not integrin β1 or α3 resulted in higher nuclear YAP levels for myoepithelial cells cultured on 30 kPa PAA gels (Extended Data Fig. 3h,j). Accordingly, integrin β4 blocking did not affect the FA length (Extended Data Fig. 3i,j). Overall, these results demonstrate that α6β4 integrins impede a rigidity-dependent increase in the nuclear localization of YAP, without affecting cellular contractility.

## α6β4 regulates mechanosensing by linking laminin to keratin

**Fig. 3:**
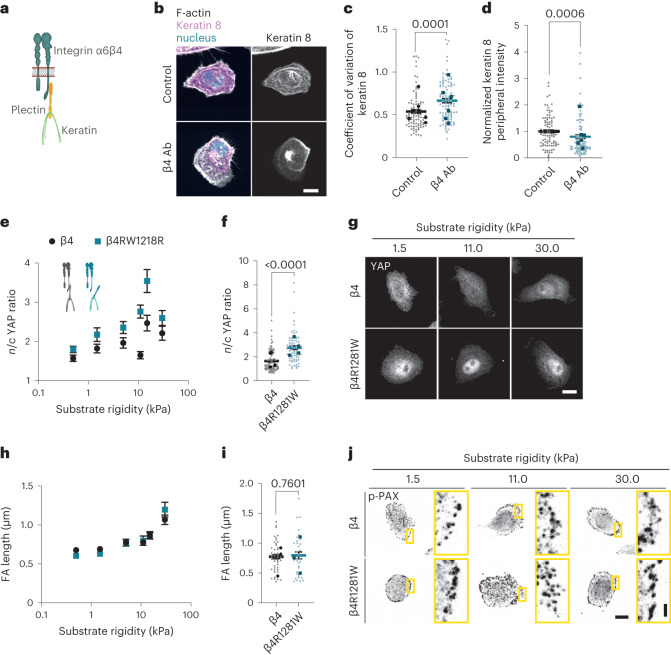
Mechanical role of integrin β4 is mediated by its connection to plectin and keratins. **a**, Schematic of the interaction of integrin α6β4 with plectin and IFs. **b**, Actin and keratin 8 stainings of MCF10A cells on 11 kPa PAA gels coated with laminin and treated with IgG (control) or integrin-β4-blocking (β4 Ab) antibodies. Scale bar, 20 μm. **c**, Quantification of the coefficient of variation of the keratin 8 signal at the basal layer of the cell periphery for control or integrin-β4-blocking (β4 Ab) conditions (unpaired two-tailed *t*-test, *n* = 88/87 cells for control or integrin-β4-blocking conditions; mean of six independent experiments). **d**, Quantification of the average mean intensity of normalized keratin 8 signal at the basal layer of the cell periphery for control or integrin-β4-blocking (β4 Ab) conditions (two-tailed Mann–Whitney test, *n* = 88/87 cells for control or integrin-β4-blocking conditions; mean of six independent experiments). **e**, Quantification of the average *n*/*c* YAP ratios of MCF10A cells overexpressing WT β4 or mutant β4RW1218R integrin seeded on PAA gels coated with laminin-111 and on different rigidities (0.5–30.0 kPa) (*n* = 69/65, 68/58, 63/65, 84/66, 51/52 and 47/66 cells for β4 and β4R1281W and increasing rigidity; mean of 4 independent experiments). The effect of both rigidity and β4 mutation is significant (*P* < 0.0001, two-way ANOVA). **f**, *n*/*c* YAP ratios at 11 kPa (two-tailed Mann–Whitney test, *n* = 84/66; mean of four independent experiments). **g**, Corresponding YAP stainings. Scale bar, 10 μm. **h**, Quantification of FA length of MCF10A cells overexpressing WT β4 or mutant β4RW1218R integrin seeded on PAA gels coated with laminin-111 and on different rigidities (0.5–30.0 kPa) (*n* = 40/33, 33/35, 31/35, 47/33, 27/19 and 36/36 cells for β4 and β4R1281W and increasing rigidity; mean of three independent experiments). The effect of rigidity is significant (*P* < 0.0001, two-way ANOVA). **i**, FA length at 11 kPa (two-tailed unpaired *t*-test, *n* = 47/33; mean of three independent experiments). **j**, Corresponding p-PAX stainings. Scale bars, 10 µm (main images)/2 μm (zoomed images). The error bars represent mean ± s.e.m.

Integrin α6β4 is a component of hemidesmosomes, which are epithelial cell contact sites linking the intermediate filament (IF) cytoskeleton to the BM^23^. This linkage is mediated by the binding of the intracellular domain of β4 to the cytolinker plectin, which, in turn, binds to IFs (Fig. 3a)^23,24^. To determine the effects on IFs, we stained for keratin 8, one of the main types of IF expressed by MCF10A cells^25^. On blocking integrin β4, the distribution of keratin 8 at the cell periphery was less homogeneous (as quantified by the coefficient of variation of the signal) and had lower intensity (Fig. 3b–d), whereas the total levels of keratin 8 intensity remained similar (Extended Data Fig. 4a). We, thus, hypothesized that since β4 does not affect the traction forces or actomyosin contraction, its role in YAP nuclear localization could be mediated by changes in keratin organization, which can indicate altered mechanical properties^26,27^.

To examine the effect of integrin β4–keratin interactions without affecting other β4 signalling functions, we introduced an integrin β4 mutant (integrin β4R1281W) that has lower affinity for plectin and therefore for IFs (Extended Data Fig. 4b,c)^24^. As a control, we transfected MCF10A cells with the wild-type (WT) version of integrin β4-GFP (Extended Data Fig. 4b,c). As expected, cells expressing mutant integrin β4R1281W lacked hemidesmosome structures (Extended Data Fig. 4d). This was observed by imaging either the transfected mutated integrin or the endogenous WT integrin. Cells expressing mutant integrin β4R1281W also had lower β4 integrin recruitment to the basal plane (Extended Data Fig. 4e,g) and a less homogeneous keratin cytoskeleton (higher coefficient of variation; Extended Data Fig. 4f,g) as a function of rigidity. Interestingly, β4 recruitment and keratin coefficient of variation did not change between collagen and laminin coatings, probably due to the binding of integrins to secreted laminin on collagen substrates (Extended Data Fig. 4h–j and Extended Data Fig. 1f). Together, these data suggest a dominant negative effect of mutation (Extended Data Fig. 4d).

We next examined the effect of integrin β4–keratin interaction on YAP. Similar to the results from blocking antibodies, MCF10A cells expressing the mutant integrin β4R1281W had a higher *n*/*c* YAP ratio that was rigidity dependent, peaking at around 11–15 kPa (Fig. 3e–g). As before, FAs and tractions were similar between the two cell types (Fig. 3h–j and Extended Data Fig. 4k–m). The mutant integrin β4R1281W also had increased YAP ratios in a multicellular monolayer setting. As with integrin-blocking experiments, this effect was more prominent at monolayer edges and on stiff versus soft substrates (Extended Data Fig. 4n–q). Further, the effect of β4R1281W integrin on both YAP and keratin 8 organization was also observed for cells seeded on laminin-332 (instead of laminin-111), although the overall YAP ratios increase with respect to laminin-111 values (Extended Data Fig. 4r–u), possibly due to the contribution of β1 integrins. Thus, the effect of β4 integrins in the nuclear localization of YAP is mediated by the anchorage between keratins and laminin substrate.

## Keratin–ECM interactions alter the cytoskeleton

To explore how laminin regulates nuclear mechanoresponses, we first hypothesized that the keratin cytoskeleton could directly affect force transmission to the nucleus, which is known to trigger YAP nuclear localization^3^, through keratin–nesprin-3 interactions. However, this was not the case (Supplementary Note 2 and Extended Data Fig. 5). Alternatively, the keratin cytoskeleton could be affecting the nucleus in a less direct way, by regulating how actin-mediated force generation reaches the nucleus. Indeed, the actin and IF cytoskeletal networks are tightly interconnected, and keratin filaments undergo retrograde flows driven by actomyosin contractility^28^. To explore how such a mechanism would operate, we implemented a computational model for the actomyosin and keratin cytoskeletons. Combined actin–IF cytoskeletal networks have been previously modelled in terms of rheology^29,30^, or to understand cell spreading^31^ or IF dynamics^32^. In our case, we used a model where IF–substrate interactions can be tuned, and that can predict force application to the nucleus and spatial distributions (as those are experimentally accessible in our setup). This can be achieved with an active gel model, commonly used for cytoskeletal networks^33^.

The model (Supplementary Note 3) considers the actomyosin cytoskeleton as an active and viscous gel undergoing turnover, in which myosin contractility leads to a continuous flow of actin towards the cell centre (retrograde flow)^34^. The keratin cytoskeleton is modelled as a passive viscoelastic gel undergoing a slower turnover compared with actin^35,36^. The actomyosin network drags the keratin cytoskeleton via internetwork friction, also leading to keratin retrograde flow. Each network is, in turn, connected to the underlying substrate through integrin-mediated adhesions, which we model with cytoskeletal–substrate friction coefficients that are spatially modulated consistently with the localization of FAs close to the cell periphery (Supplementary Fig. 1b).

Since integrin β4 binds to the keratin cytoskeleton as well as the surrounding substrate, it can be understood as a ‘docking site’ for the keratin network to the substrate. This incorporation will have two effects. First, it will decrease the ability of actomyosin retrograde flow to drag the keratin network. We incorporate this into the model by lowering the friction coefficient between the keratin network and substrate (*η*^IF^) in the case of the integrin β4R1281W mutant due to the absence of stable integrin β4–keratin connections (Supplementary Fig. 1b). Second, the incorporation of the keratin network into hemidesmosomes will increase the crosslinking of keratin filaments, which, in turn, will stiffen the keratin network^31,37^. Since, in our model, keratin–substrate friction accounts for the mechanical effect of hemidesmosomes, we included this effect by increasing the elastic modulus of the IF network *G* proportionally to the keratin–substrate friction *η*^IF^.

**Fig. 4:**
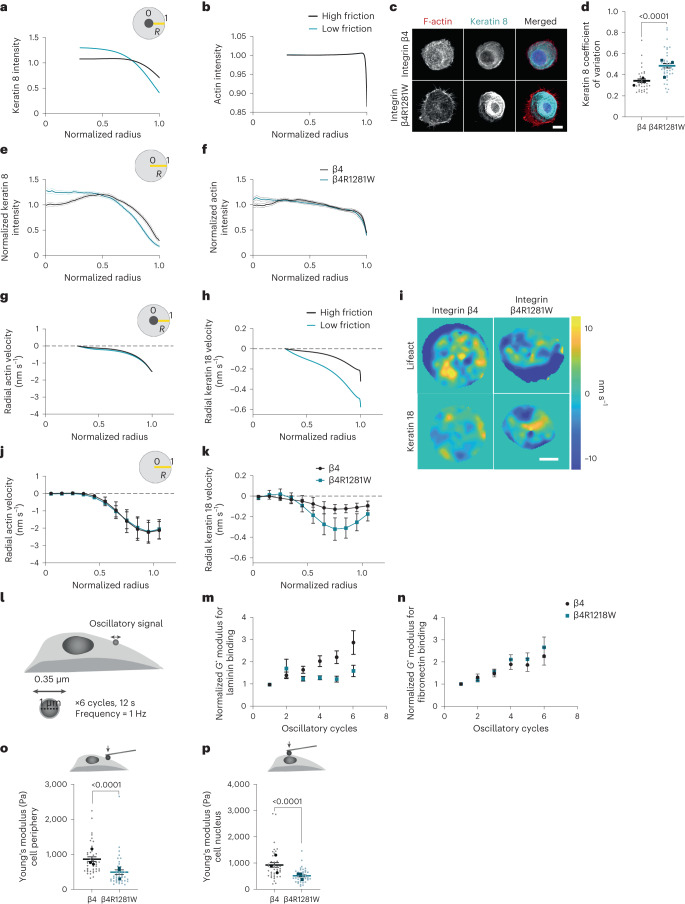
Laminin–integrin β4–keratin link regulates cytoskeletal dynamics and cell mechanical properties. **a**, Model predictions for keratin distribution along the cell radius (*R* = 1, cell periphery; *R* = 0, cell centre) for high and low keratin–substrate friction (*η*_0_^IF^ of 8 versus 2 kPa s µm^–2^). Predictions start at *R* = 0.33, where the nucleus edge is assumed to be present. **b**, Model predictions for actin distribution along the cell radius. **c**, Phalloidin (F-actin) and keratin 8 stainings of MCF10A cells overexpressing WT β4 or mutant β4RW1218R integrin, seeded on circular laminin patterns of 30 μm diameter on 12 kPa PDMS substrates. Scale bar, 10 μm. **d**, Corresponding quantification of coefficient of variation of keratin 8 signal at the basal layer of the cell periphery (two-tailed unpaired *t*-test, *n* = 35/40 for β4/β4R1281W; mean of three independent experiments). **e**, Normalized keratin 8 intensity along the cell radius (*R* = 1, cell periphery; *R* = 0, cell centre) (*n* = 27/43 cells for β4/β4R1281W; mean of three independent experiments). The combined effect of β4 mutation and radial distribution of keratin intensity is significant (*P* < 0.0001, two-way repeated-measures ANOVA). **f**, Normalized phalloidin signal intensity along the cell radius (*n* = 27/43 cells for β4/β4R1281W; mean of three independent experiments). The combined effect of β4 mutation and radial distribution of keratin intensity is significant (*P* < 0.0001, two-way repeated-measures ANOVA). **g**, Model prediction of radial retrograde flows of actin along the cell radius. **h**, Model prediction of radial retrograde flows of keratin along the cell radius. **i**, Snapshot colour maps of Lifeact-GFP (top) or keratin 18-mCherry (bottom) velocities (nm s^–1^) as calculated by PIV. Scale bar, 10 μm. **j**, Experimental actin retrograde flows along the cell radius (*n* = 12 cells for both β4 and β4R1281W). The combined effect of β4 mutation and radial actin velocities is not significant (*P* = 0.6927, two-way repeated-measures ANOVA). **k**, Experimental keratin 18 retrograde flows along the cell radius (*n* = 12 cells for both β4 and β4R1281W). The combined effect of β4 mutation and radial keratin velocities is significant (*P* = 0.0296, two-way repeated-measures ANOVA). **l**, Optical tweezers setup. **m**, Bead resistance to force (shear modulus G*) for β4- or β4R1281W-overexpressing cells seeded on laminin-coated glass coverslips on oscillatory stimulation with laminin-coated beads (*n* = 38/32 for β4/β4R1281W cells, mean of three independent experiments). The effect of β4 mutation is significant (*P* = 0.0110) and the effect of the number of oscillatory cycles is significant (*P* = 0.0054, paired mixed-effects model (REML)). **n**, Evolution of G* moduli for β4- or β4R1281W-overexpressing cells seeded on laminin-coated glass coverslips on oscillatory stimulation with fibronectin-coated beads (*n* = 30/36 for β4/β4R1281W cells, mean of three independent experiments). The effect of β4 mutation is not significant (*P* = 0.5988) and the effect of the number of oscillatory cycles is significant (*P* < 0.0001, paired mixed-effects model (REML)). **o**, Young’s modulus of the cell cytoplasm of MCF10A cells overexpressing WT β4 or mutant β4RW1218R integrin seeded on laminin-coated glass coverslips (unpaired two-tailed *t*-test, *n* = 41/40 for β4/β4R1281W cells; mean of three independent experiments). **p**, AFM stiffness measurements above the cell nucleus of MCF10A cells overexpressing WT β4 or mutant β4RW1218R integrin seeded on laminin-coated glass coverslips (unpaired two-tailed *t*-test, *n* = 40/40 for β4/β4R1281W cells; mean of three independent experiments). The error bars represent mean ± s.e.m.

The model predicts that actomyosin flows drag keratin more efficiently in mutant cells due to decreased friction with the substrate. This will lead to keratin accumulation around the cell nucleus, whereas the organization of actin will remain largely unaffected due to its higher turnover rate (Fig. 4a,b). To validate this prediction, we seeded cells on round laminin patterns of 30 μm diameter (Fig. 4c). We first assessed the keratin network homogeneity by calculating the coefficient of variation of the keratin 8 signal at the cell periphery. This showed an increase for β4R1281W- versus WT-integrin-expressing cells (Fig. 4d), indicating a disrupted keratin organization, and confirming our previous observations with integrin-β4-blocking antibodies (Fig. 3c). We then calculated the keratin distribution across the cell radius, where *R* = 1 is the cell periphery as marked by actin staining and *R* = 0 is the cell centre (Fig. 4e). Results were in marked agreement with the model predictions, where keratin 8 had a more central localization in integrin-β4R1281W-expressing cells (Fig. 4e and Extended Data Fig. 6a). Accordingly, actin had a more homogeneous distribution throughout the cell in both conditions (Fig. 4f and Extended Data Fig. 6b).

A second set of predictions from the model is that both actin and keratin should exhibit retrograde flows, keratin flows should generally be lower than actin flows and keratin flows should be higher in mutant-integrin-β4-expressing cells due to reduced friction (Fig. 4g,h). To test these predictions, we used the same circular patterns and performed the time-lapse imaging of control or mutant integrin β4 cells transfected with Lifeact-GFP or keratin 18-mCherry (the binding partner of keratin 8). Then, we applied particle image velocimetry (PIV) to calculate the actin and keratin 18 flows (Fig. 4i and Supplementary Videos 1 and 2). The experimental values and trends for retrograde flows (measured through radial velocities) were in agreement with the model predictions (Fig. 4j,k; Supplementary Note 4 provides an extended discussion).

## A stable laminin–keratin link increases cellular stiffness

A major assumption of our model is that modulating the laminin–keratin link through β4 integrins should affect keratin crosslinking and therefore the stiffness of the keratin network^31^. In turn, this should affect the cell mechanics, as the keratin network is the main cytoskeletal component contributing to the mechanical integrity of most epithelial tissues^38^. To assess this, we coated 1 µm silica beads with laminin-111, placed them in contact with the cell surface until they attached and then horizontally oscillated the beads with optical tweezers (Fig. 4l). On oscillation, the mechanical resistance of the cytoskeleton gradually increased, as quantified by the complex shear modulus G* (Fig. 4m). Consistent with our hypothesis, this increase in the mechanical resistance was largely abrogated for cells transfected with mutant integrin β4R1281W (Fig. 4m). Confirming the specific role of laminin–integrin β4 links, the differences between WT and R1281W integrin β4 were lost when beads were coated with fibronectin instead of laminin, which binds to integrins other than β4 (ref. ^34^) (Fig. 4n). Further, cell stiffness as measured with atomic force microscopy (AFM) was significantly higher in cells transfected with WT compared with R1281W integrin β4, both at the cell periphery and above the cell nucleus (Fig. 4o,p). Thus, direct local force application to laminin–integrin β4–keratin connections increases the mechanical resistance of the cytoskeleton, and a stable connection of keratins with the laminin substrate increases the cytoskeletal stiffness.

## Keratin protects nuclei from actin-mediated deformation

**Fig. 5:**
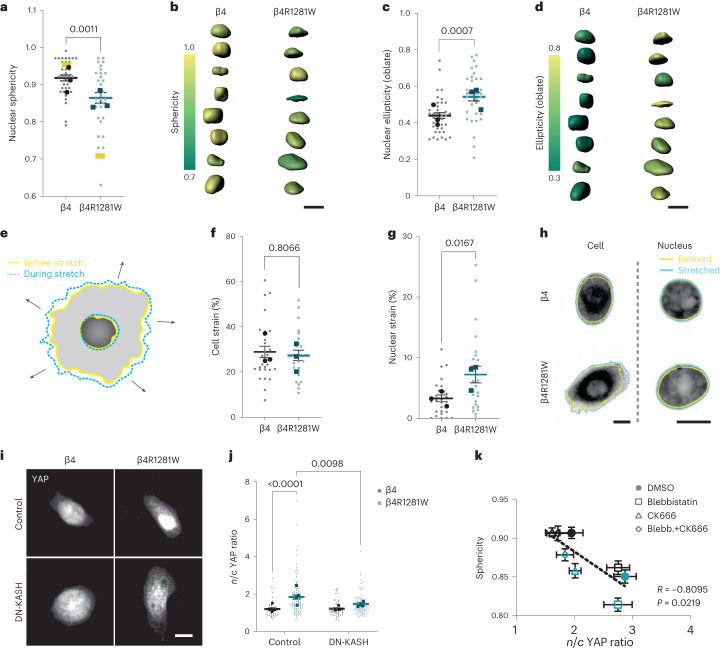
Laminin–integrin β4–keratin link shields the nucleus from actin-mediated deformation. **a**, Nuclear sphericity measurements of MCF10A cells overexpressing WT β4 or mutant β4RW1218R integrin seeded on 11 kPa PAA gels coated with laminin (unpaired two-tailed *t*-test, *n* = 37/32 for β4-/β4R1281W-overexpressing cells; mean of three independent experiments). The yellow bars show the model predictions (for high and low keratin–substrate frictions, *η*_0_^IF^ of 8 versus 2 kPa s µm^–2^). **b**, Three-dimensional segmentation and sphericity of the colour-coded nuclei for cells overexpressing integrin β4 (left) and β4R1281W (right). Scale bar, 10 μm. **c**, Nuclear ellipticity (oblate) measurements (two-tailed unpaired *t*-test, *n* = 37/32 for β4-/β4R1281W-overexpressing cells; mean of three independent experiments). **d**, Three-dimensional segmentation and ellipticity (oblate) of the colour-coded nuclei for cells transfected with integrin β4 (left) and β4R1281W (right). Scale bar, 10 μm. **e**, Illustration of cell and nuclear strain on stretching. **f**, Cellular strain on an equiaxial stretching of MCF10A cells overexpressing WT β4 or mutant β4RW1218R integrin seeded on laminin-coated PDMS membranes (two-tailed Mann–Whitney test, *n* = 27/24 for β4/β4R1281W; mean of three independent experiments). **g**, Nuclear strain of MCF10A cells overexpressing WT β4 or mutant β4RW1218R integrin seeded on laminin-coated PDMS membranes (two-tailed Mann–Whitney test, *n* = 27/24 for β4/β4R1281W; mean of three independent experiments). **h**, Examples of cell (marked with CellTracker) and nuclear (marked with Hoechst 33342) strain for integrin-β4- and β4R1281W-overexpressing cells. Scale bar, 10 μm. **i**, Control or DN-KASH-transfected cells stained for YAP. Representative images of cells seeded on laminin-coated 11 kPa gels. Scale bar, 10 μm. **j**, *n*/*c* YAP ratios for control or DN-KASH-transfected integrin β4 and β4R1281W cells seeded on laminin-coated 11 kPa gels (control (*n* = 48/88) and DN-KASH (*n* = 63/76) for β4/β4R1281W; mean of four independent experiments, two-way ANOVA, Bonferroni correction for multiple comparisons). **k**, *n*/*c* YAP ratios and sphericity correlation for cells expressing WT β4 (black) or β4R1281W (blue) treated with different drugs (*R* is Spearman’s correlation coefficient). The error bars represent mean ± s.e.m.

Once the overall effects on the cytoskeleton were established, we assessed how those would affect the nucleus. We and others have previously shown that force transmission to the nucleus through the actin cytoskeleton can deform the nucleus, leading to YAP nuclear entry^3,39^. We, therefore, assessed if differences in the mechanical deformation of the nucleus could explain the altered nuclear mechanoresponses of mutant integrin β4 cells. We first calculated the model predictions of applied mechanical tension to the nucleus, which showed that the nucleus is under tension from myosin pulling forces. Due to the reduced friction with keratin, this tension is transmitted more effectively in mutant-β4R1281W-expressing cells, increasing by ~1.6% (Extended Data Fig. 7a). Assuming the same nuclear stiffness in WT-β4- and β4R1281W-expressing cells, this difference in tension would only result in a small nuclear deformation, as quantified by a reduction in the sphericity of the nucleus of less than 1%. However, our data show decreased cell stiffness in mutant β4R2181W cells (Fig. 4o,p), attributed to lower crosslinking of the keratin network. Accounting for such a reduction in the nuclear stiffness, our model predicts a more pronounced increase in nuclear deformation in mutant cells, as quantified by the lower sphericity (Fig. 5a, yellow bars). Due to this combined effect, the keratin–substrate friction parameter is interestingly the one that has the largest effect on nuclear shape, even more than myosin contractility (Supplementary Note 3). This suggests that modulating the keratin–ECM attachment may indeed be a very effective way to regulate the nuclear mechanics.

We then experimentally measured the nuclear shapes for WT-integrin-β4 and β4R1281W-expressing cells seeded on 11 kPa PAA gels, where the differences in nuclear YAP levels were the highest. Nuclear shapes were markedly different between the cell lines, and sphericity was lower in mutant cells (Fig. 5a,b). Although experimental differences were somewhat smaller than model predictions (possibly due to contributions of factors other than keratins to the nuclear mechanical properties), the trends agreed, and predictions fell within the experimental range of values. Overall, mutant β4R2181W cells were less spherical because they were more oblate (Fig. 5c,d) and therefore more flattened in the horizontal dimension. WT-β4-expressing cells resembled a more prolate ellipsoid, and overall, the degree of oblateness/prolateness positively/negatively correlated with nuclear YAP (Extended Data Fig. 7b–e). No changes in nuclear volume were observed between the two cell types (Extended Data Fig. 7f).

Then, we explicitly evaluated whether the nuclei in β4R1281W-integrin-transfected cells had decreased mechanical shielding. To this end, we stretched the cells using a custom-built stretching device^40^ and measured the cellular and nuclear strain (Fig. 5e). Cells transfected with either WT or β4R1281W integrins stretched to the same degree (Fig. 5f,h). However, the cell nucleus stretched less in WT-transfected cells than in R1281W-β4-transfected cells (Fig. 5g,h), confirming that the laminin–keratin link protected the nucleus from mechanical deformation. Finally, we verified whether our observed effects in YAP nuclear localization were indeed explained by changes in actin-mediated force transmission to the nucleus. We co-transfected cells with either WT or R1281W integrin β4 and DN-KASH, the KASH domain of nesprin-1. This domain binds to its binding partner in the nuclear lamina protein SUN, acting as a dominant negative that prevents endogenous nesprin-1 and nesprin-2 binding. This disrupts the linker of the nucleoskeleton and cytoskeleton complex, preventing force transmission from the actin cytoskeleton to the nucleus^41^. DN-KASH expression decreased the YAP *n*/*c* ratios in R1281W-β4-transfected cells, bringing them down to levels similar to WT-transfected cells (Fig. 5i,j), thereby confirming the role of actin-mediated force transmission to the nucleus. Confirming this role, we found a tight correlation between nuclear shapes and YAP *n*/*c* ratios after different drug treatments (Fig. 5k, Extended Data Fig. 7g–i and Supplementary Note 5). Our findings, thus, draw a mechanism where the laminin–keratin links shield the nucleus from actin-mediated mechanical deformation.

## Additional effects of laminin–keratin nuclear shielding

Apart from nuclear YAP translocation, forces applied to the nucleus can trigger different mechanotransduction events, which could also take place in our system. For instance, nuclear deformation regulates the lamin A/C levels^42^. We found no significant difference between the WT and β4R1281W cells regarding lamin A/C expression, indicating that the nuclear lamina was not compromised (Extended Data Fig. 8a–c). Further, sustained mechanical strain has been reported to increase H3K27me3, a histone mark indicative of compacted heterochromatin^43^. Consistently, cells expressing mutant integrin β4R1281W had higher levels of this heterochromatin mark compared with WT-expressing cells (Extended Data Fig. 8d,e). This does not provide conclusive evidence on a general effect on chromatin compaction, which would require a more in-depth study. However, it shows that the mechanical shielding of the nucleus provided by the laminin–keratin link impairs not only YAP but also other reported effects of nuclear mechanotransduction.

**Fig. 6:**
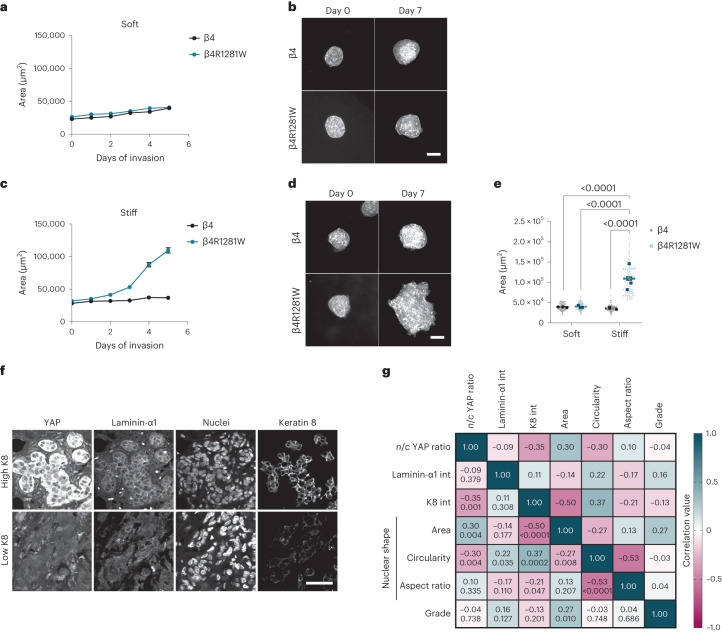
Keratin-mediated mechanoprotection prevents three-dimensional cell invasion and can also be observed in vivo. **a**, Area quantifications of MCF10A cell spheroids overexpressing WT β4 or mutant β4RW1218R integrin over 5 days in soft alginate–Matrigel matrices (Young’s modulus, 1.5 kPa; *n* = 90 spheroids; mean of three independent experiments). **b**, Sample phalloidin images of MCF10A cell spheroids overexpressing WT β4 or mutant β4RW1218R integrin in soft alginate–Matrigel matrices. Scale bar, 100 μm. **c**, Area quantifications of MCF10A cell spheroids overexpressing WT β4 or mutant β4RW1218R integrin over 5 days in stiff alginate–Matrigel matrices (Young’s modulus, 4.5 kPa; *n* = 90 and 90/90/89/89/88 for β4 or β4R1281W spheroids; mean of three independent experiments). **d**, Sample phalloidin images of MCF10A cell spheroids overexpressing WT β4 or mutant β4RW1218R integrin in stiff alginate–Matrigel matrices. Scale bar, 100 μm. **e**, Integrin β4 and β4R1281W cell spheroids on day 5 of invasion (*n* = 90/90 and 90/88 for β4/β4R1281W and soft or stiff matrices; mean of three independent experiments). Both effect of stiffness and β4 mutation are significant (*P* < 0.0001, two-way ANOVA, Bonferroni correction for multiple comparisons). **f**, Tissue sections of breast cancer tumour samples expressing low and high levels of keratin 8. Scale bar, 50 μm. **g**, Pearson’s correlation matrix of tumour samples (*n* = 93 tumour areas from 31 different tumour samples). The correlations are indicated in bold, and the *P* values are shown in italic.

Finally, we assessed the potential involvement of this mechanism in cancer invasion. First, we seeded cells as spheroids, inside a three-dimensional Matrigel (rich in laminin-111) crosslinked with alginate, as previously described^5,44^, to regulate stiffness without affecting the ligand density. As expected and previously described^5^, MCF10A cells expressing WT β4 integrin were not invasive, regardless of the matrix stiffness. Mutant cells, however, remained non-invasive on soft gels (Young’s modulus, 1.5 kPa), but were invasive on a stiffer matrix (4.5 kPa) (Fig. 6a–e). The detailed mechanisms driving invasion in this setting remain to be understood and may not be necessarily related to YAP (which remained cytosolic in these experiments; Extended Data Fig. 8f,g). However, the experiments show that changes in cell mechanical properties by abrogating the keratin–ECM link and its mechanoprotective role promote invasion.

Second, we quantified the *n*/*c* YAP ratios, laminin-α1 and keratin 8 intensities and nuclear shapes in human invasive carcinomas. The YAP levels, keratin intensities and nuclear shapes showed statistically significant correlations, in line with our observations in vitro (Fig. 6f,g). Correlations with laminin were weaker but followed the same trends as keratin, as expected. The weaker correlation with laminin-α1 may be due to the presence of other matrix components, which may override the laminin response (Extended Data Fig. 1g–i).

## Discussion

Our results demonstrate a context-specific mechanoresponse, where laminin-111 impairs the cellular response to substrate rigidity. We demonstrated this mechanism in vitro but provide the evidence of its potential role in vivo. Our mechanism could help interpret several previous results in the literature, both in vitro and in vivo. Loss of laminin-111 expression has been correlated with breast cancer progression^13^. However, in ductal carcinoma in situ where the laminin levels may still be uncompromised, YAP was not nuclear despite changes in the rigidity of the tissue, and nuclei appeared smaller and rounder compared with invasive ductal carcinoma models that expressed a subset of YAP target genes^7^. Moreover, the binding of integrin α6β4 to the BM prevented the stiffness-induced malignant phenotype of mammary epithelial cells^5^, and blocking integrin β4 triggered a malignant phenotype in an in vitro breast cancer model^11^. Finally, cell softening in the invasive front of mammary epithelial spheroids has been associated with changes in cell and nuclear shapes, and a differential expression of keratins, supporting an association between the keratin cytoskeleton and cell stiffness and invasiveness^45,46^. Indeed, changes in keratin expression during breast cancer invasion (that is, from a luminal keratin 8 to a basal keratin 14) (ref.^46^) could also affect the mechanical properties and responsiveness of the keratin network to external signals.

We, thus, propose that cell engagement to laminin, and its subsequent effect on nuclear and cell mechanics through the keratin cytoskeleton, could affect tissue organization and cell responses. Loss of these interactions could promote a malignant phenotype by rendering the nuclei more susceptible to deformation. These context-specific nuclear mechanoresponses could possibly explain the often contradicting roles of YAP in breast cancer progression^7,47,48^, or the opposing roles of myosin contractility in YAP nuclear localization in different cell types^22,49^. Similarly, α6β4 integrins have been reported to both induce and inhibit malignant phenotypes^50,51^. The tumour-promoting role of α6β4 involves contexts probably unrelated to mechanical effects on keratins, as it is maintained for cells in suspension^52^ or lacking the extracellular domain of α6β4 (refs. ^53,54^), or involves a switch of α6β4 from keratin to actin binding^55^.

IFs are largely considered as the stronghold of cell and tissue integrity. Recently, both keratin and vimentin networks have been shown to provide mechanical support to the cell nucleus^27,56^. This mechanical role of IFs is regulated by several biochemical signals, such as phosphorylation or divalent cations^38^. Here we show that mechanical protection by IFs can also be tuned by ECM composition and stiffness, which can have implications for chromatin methylation. Thus, changes in the ECM could eventually lead to altered gene expression and differentiation. For instance, repeated cycles of mechanical strain in epidermal progenitor cells led to a decrease in nuclear actin levels and a force-driven perinuclear actin polymerization, resulting in H3K27me3-mediated gene silencing, eventually attenuating lineage commitment^57^. Responses of this kind may be modulated through mechanical protection by keratins. This could be relevant in the many scenarios where laminin and keratin play a role, from cancer to the very early stages of embryonic development and cell differentiation events^58–60^.

## Methods

### Cell culture

Mammary epithelial cells (MCF10A) were purchased from ATCC (category no. CRL-10317). Cells were used for a maximum of 18 passages and were cultured in DMEM-F12 (Life Technologies, 21331-020) with 5% horse serum, 1% penicillin–streptomycin, epidermal growth factor (20 ng ml^−1^), hydrocortisone (0.5 μg ml^−1^), cholera toxin (100 ng ml^−1^) and insulin (10.0 μg ml^−1^). Human-breast-myoepithelial-immortalized cell lines were obtained from J. Louise Jones (Barts Cancer Institute, Queen Mary University London), as described previously^61,62^, and used for a maximum of eight passages. They were cultured in Ham’s-F12 (Sigma, N4888) media supplemented with 10% foetal bovine serum, 1% penicillin–streptomycin, hydrocortisone (1 μg ml^−1^), epidermal growth factor (10 ng ml^−1^) and insulin (5 μg ml^−1^). All the cells were regularly tested for mycoplasma contamination. HEK293T cells for retroviral production were a gift from N. Montserrat (Institute for Bioengineering of Catalonia).

### Preparation of PAA gels

PAA gels were prepared as described previously^62^. Briefly, glass-bottom MatTek dishes and slides were activated with a solution of acetic acid, 3-(trimethoxysilyl)propyl methacrylate (Sigma) and 96% ethanol (1:1:14) for more than 10 min and 2 h for the glass-bottom MatTek dishes and glass slides, respectively. The glass was then washed with 96% ethanol and air dried. Different concentrations of acrylamide and bis-acrylamide were mixed to produce gels of different rigidities^4^ and mixed together with 2.00% v/v fluorescent carboxylated 200 nm beads (Invitrogen), 0.50% APS (A3678, Sigma) and 0.05% v/v tetramethylethylenedi-amine (T9281, Sigma). The solution was placed on the glass and covered with a coverslip, letting the gel to set for 50 min. The coverslip was then removed, and the gels were coated with 50 μl of 10.0% v/v 0.5 M HEPES (pH 6.0), 2.0% v/v of 0.2% bis-acrylamide, 1.0% v/v Igracure 2959 and 4.0% v/v of 10 mg ml^–1^ *N*-hydroxysuccinimide (Sigma-Aldrich) in dimethyl sulfoxide (DMSO, Sigma-Aldrich). The gels were then exposed to ultraviolet light (XX-15, UVP) at 365 nm wavelength for 5 min, washed twice with a 0.5 M HEPES (pH 6.0) solution followed by 2× PBS solution washes. The gels were then incubated overnight at 4 °C with 10 μg ml^–1^ (unless specified otherwise; Extended Data Fig. 1) solution of laminin-111 (L2020, Sigma), collagen I (First Link (UK)), fibronectin (F0895, Sigma-Aldrich) and laminin-332 (LN332-0502, BioLamina) protein solution in PBS. The rigidity of PAA gels was measured and characterized with AFM, as described previously^62^. Before cell seeding, the gels were incubated with a serum-free medium for 30 min. The cells were then seeded on gels in the same medium and fixed after 6 h. For monolayer experiments, the cells were fixed 24 h post-seeding. For integrin β4 expression levels (Extended Data Fig. 3g) and lamin A/C and H3K27me3 experiments (Extended Data Fig. 8), the cells were fixed after 16 h to allow time for differences in the protein-level expression.

Protein quantification on gels was performed by placing known amounts of proteins (laminin, collagen I and fibronectin) on polydimethylsiloxane (PDMS), which was then dried, fixed, blocked with 1% bovine serum albumin in PBS and stained using 1:200 rabbit anti-laminin (ab11575, Abcam), anti-collagen I (AB755P, Millipore), anti-fibronectin (F3648, Sigma) and 1:500 donkey 488 anti-rabbit secondary (A-21206, Thermo Fisher). A standard curve was generated by correlating fluorescence intensity and the amount of protein. At the same time, PAA gels of 1.5, 11.0 and 30.0 kPa were coated with 10 μg ml^–1^ laminin, collagen I and fibronectin, as described before, and stained the same way as that for the standard curve of the corresponding protein. Epifluorescence images of the standard curve and gel samples were acquired using a ×2 objective in an inverted microscope (Nikon ECLIPSE Ti) using MetaMorph (NIS Elements) imaging software. The average amount of protein per square millimetre was calculated according to the intensity of the signal of the gels. For molar calculations, the molecular weight used for laminin-111, collagen I and fibronectin was 900, 200 and 300 kDa, respectively.

### Immunostaining

For immunostaining, the cells were fixed with 4.0% paraformaldehyde for 10 min, permeabilized with 0.1% Triton X-100 for 4 min; after a 30 min blocking step with 0.5% fish gelatin, the cells were incubated with primary antibodies (1.5 h, room temperature in 0.5% fish gelatin in PBS), followed by incubation with secondary antibodies. When phalloidin-Atto 488 (Sigma-Aldrich, category no. 49409), phalloidin-TRITC (Sigma-Aldrich, category no. P1951) and phalloidin-iFluor 647 (Abcam, category no. ab176759) reagents were used, they were added with the secondary antibodies. Hoechst 33258 staining dye was used for nuclear labelling following 10 min incubation at room temperature. For the stainings of ECM protein secretion (Extended Data Fig. 1f), no permeabilization was performed to identify only the secreted amounts of protein.

The immunostaining of three-dimensional alginate hydrogels was performed by fixing the hydrogels with 4% paraformaldehyde for 30 min. After fixation, the cells were permeabilized and blocked with 0.5% Triton and 3.0% goat serum in PBS with calcium (blocking buffer) for 12 h. Once the hydrogels were permeabilized and blocked, primary antibody anti-YAP1 (Santa Cruz, 101199, 1:200) was added in a blocking buffer for 24 h. After incubation with the primary antibody, the hydrogels were washed five times for 24 h in a blocking buffer. Next, the gels were incubated in Alexa Fluor 555 secondary antibody (Thermo Fisher, A32727, 1:500), Alexa Fluor 647 phalloidin (Thermo Fisher, A22287, 1:500) and Hoechst 33342 (Thermo Fisher, 62249, 1:2,000) in a blocking buffer for 12 h. Afterwards, the gels were washed in PBS with calcium overnight. Finally, ProLong antifade mountant (Thermo Fisher, P36930) was added to the hydrogels before imaging.

The primary antibodies used and their respective dilutions are as follows: rabbit p-Pax 1:100 (Tyr118) (Cell Signaling, category no. 69363 and 2541s), rabbit anti-YAP (D8H1X) XP 1:100 (Cell Signaling, category no. 14074), mouse anti-YAP1 (63.7) 1:100 (Santa Cruz, category no. sc-101199), rabbit anti-cytokeratin 8 (EP1628Y) 1:200 (Abcam, category no. ab53280), rabbit anti-plectin antiserum 1:400 (#46, gift from G. Wiche), mouse anti-integrin β4 (M126) 1:1,000 (Abcam, category no. ab29042), mouse anti-lamin A + lamin C antibody (131C3) 1:200 (Abcam, category no. ab8984), mouse anti-lamin A/C (E1) 1:100 (Santa Cruz, category no. sc376248), rabbit anti-tri-methyl-histone H3 (Lys27) (C36B11) 1:300 (Cell Signaling, category no. 9733), rabbit anti-laminin 1:200 (Abcam, category no. ab11575), anti-collagen I 1:200 (Millipore, category no. AB755P), anti-fibronectin 1:200 (Sigma, category no. F3648), rabbit anti-vimentin 1:250 (Abcam, category no. ab92547).

The secondary antibodies used are as follows: mouse Alexa Fluor 488 (category no. A-110229), Alexa Fluor 555 (category no. A-21424 and A-31570), Alexa Fluor 647 (category no. A-21236) and rabbit Alexa Fluor 488 (category no. A-21206), Alexa Fluor 555 (category no. A-21429), Alexa Fluor 647 (category no. A-21245); all of these were used at 1:300 concentration (Thermo Fisher).

### Image acquisition

Immunofluorescence images and actin/keratin retrograde flow experiments were performed in a Nikon TiE inverted microscope with a spinning-disc confocal unit (CSU-WD, Yokogawa) and a Zyla scientific complementary metal–oxide–semiconductor camera (Andor) with μManager (version 1.4.22), using a ×60 objective (Plan Apo; numerical aperture (NA), 1.2; water-immersion type). Epifluorescence images were taken on an automated inverted microscope (Nikon ECLIPSE Ti) using MetaMorph (NIS Elements) imaging software (version 7.7.10) and a ×60 objective (Plan Apo VC; NA, 1.4; oil-immersion type). Higher-resolution confocal images (Extended Data Fig. 4) and three-dimensional segmentation of nuclei (Fig. 5 and Extended Data Fig. 7b–f) were acquired using a ZEISS LSM 880 inverted confocal microscope with Airyscan and a ×63 1.46-NA oil-immersion objective and ZEN (ZEISS, version 2.3 SP1 FP3 black) software. Spheroids were imaged using an inverted ZEISS LSM 880 confocal microscope with an oil-immersion ×40 objective with an NA of 1.3.

### Immunostaining analysis

**Extended Data Fig. 2:**
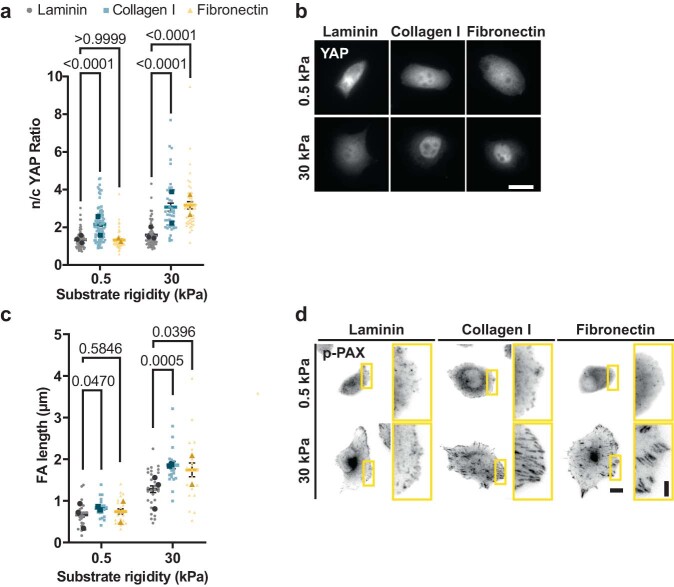
Rigidity response of myoepithelial cells on different substrates. **a** Quantification of n/c YAP ratio (*n* = 82/84/61, 70/43/48 cells for laminin/collagen I/fibronectin for 0.5 and 30 kPa respectively; mean of at least 2 independent experiments). The effect of both rigidity and substrate coating is significant. (*P* < 0.0001, two-way ANOVA, Bonferroni correction for multiple comparison). **d** Sample YAP images for human myoepithelial cells on laminin/collagen I/fibronectin substrates of 0.5 and 30 kPa stiffness; scale bar is 20 μm. **c** Quantification of FA length from phospho-paxillin (p-Pax) imaging (*n* = 24/20/20, 28/25/20 cells for laminin/collagen I/fibronectin for 0.5 and 30 kPa respectively; mean of at least 2 independent experiments). The effect of both rigidity (*P* < 0.0001) and substrate coating (*P* = 0.0002) is significant (two-way ANOVA, Bonferroni correction for multiple comparison). **d** Sample p-Pax images for human myoepithelial cells on laminin/collagen I/fibronectin substrates of 0.5 and 30 kPa stiffness; scale bars are 10 μm (main images)/ 4 μm (zoomed images). Error bars represent mean ± s.e.m.

**Extended Data Fig. 3:**
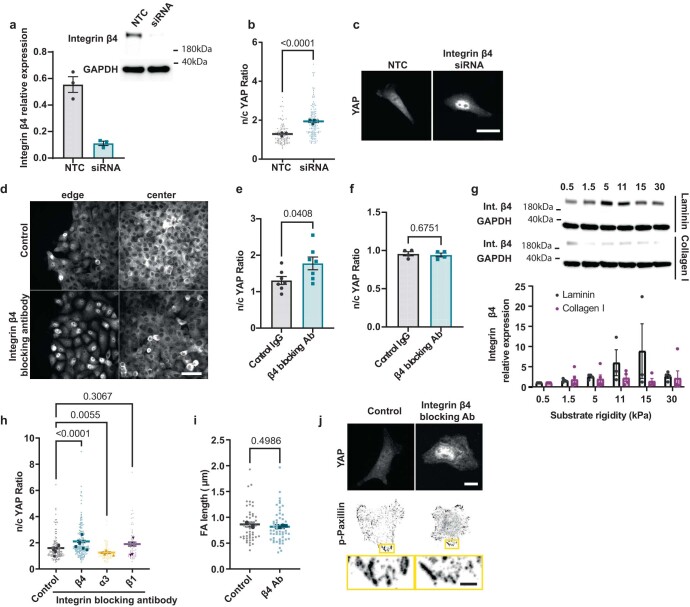
Further experiments on the role of α6β4 integrins. **a** Western blot for cells transfected with a control non-targeting siRNA (NTC) or integrin β4 siRNA (siRNA) and quantification (n = 3 independent experiments). **b** n/c YAP ratio of NTC or integrin β4 siRNA transfected cells seeded on 11 kPa laminin-coated PAA gels (two-tailed Mann-Whitney test, n = 124/148 from 3 independent experiments). **c** Sample YAP stainings of NTC or integrin β4 siRNA transfected cells; scale bar is 20 μm. **d** YAP stainings of MCF10A cell colonies treated with control IgG/β4 blocking antibodies for cells grown on 11 kPa laminin-coated PAA gels. Left panel shows cells at the edge of the monolayer and on the right corresponding images of the monolayer centre; scale bar 50μm. **e** Quantification of n/c YAP ratios of MCF10A cell colonies seeded on 11 kPa laminin-coated PAA gels at the edge of the monolayer upon treatment with control IgG or integrin β4 blocking antibody (unpaired two-tailed t-test, n = 7/7 colonies). **f** Quantification of n/c YAP ratios of MCF10A cell colonies seeded on 11 kPa laminin-coated PAA gels at the centre of the monolayer upon treatment with control IgG or integrin β4 blocking antibody (unpaired two-tailed t-test, n = 4/4 colonies). **g** Western blot for integrin β4 expression levels of MCF10A cells on laminin or collagen I coated gels of different rigidities, quantification of at least 3 independent experiments. **h** Average values of nuclear/cytosolic YAP ratio of myoepithelial cells seeded on laminin coated PAA gels of 30 kPa stiffness upon treatment with control or integrin β4/α3/β1 blocking antibodies (*n* = 145/165/76/61 cells for control/β4/α3/β1; mean of at least 3 independent experiments, *P* < 0.0001, Kruskal-Wallis test, Dunn’s correction for multiple comparison). **i** Focal adhesion length from p-PAX stainings of control and integrin β4 blocking antibody treated myoepithelial cells seeded on laminin coated PAA gels of 30 kPa stiffness (n = 48/55; mean of 3 independent experiments, two-tailed unpaired t-test). **j** Corresponding images of YAP and p-Pax stainings for control and integrin β4 blocking antibody treated myoepithelial cells; scale bars are 10 μm (main images)/ 2.5 μm (zoomed images). Error bars represent mean ± s.e.m. Source data

**Extended Data Fig. 4:**
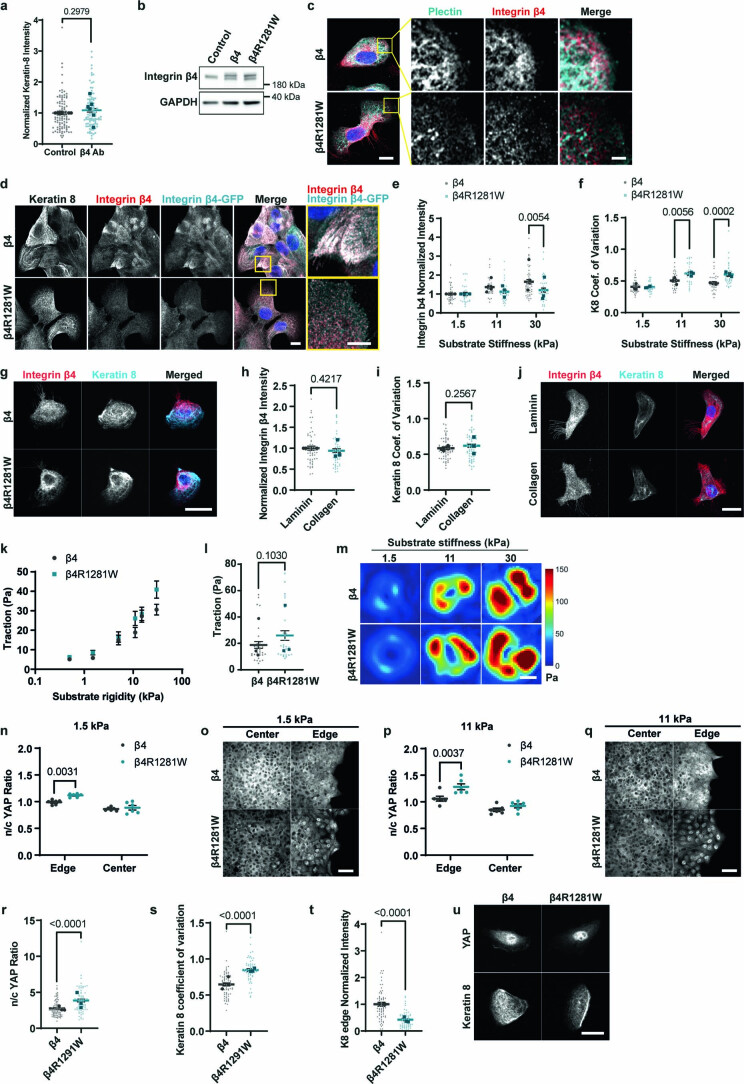
Further characterization of the effects of keratin-laminin links. **a** Normalized mean intensity of sum confocal projections of keratin 8 stainings of cells seeded on 11 kPa laminin-coated PAA gels (unpaired two-tailed t-test, n = 93/89 cells for control or integrin β4 blocking conditions (β4 Ab); mean of 6 independent experiments). **b** Western blot of integrin β4 in MCF10A cells in control conditions or after overexpressing WT or β4R1281W β4 in MCF10A cells. **c** Stainings for plectin (cyan) and integrin β4 (red) in cells overexpressing WT integrin β4 or β4R1281W integrin β4 seeded on laminin-coated glass coverslips; scale bars 10 µm (main images) and 2 μm (zoomed images). **d** Stainings for keratin 8 (grey), integrin β4 (total, red) and GFP (overexpressed integrin β4, cyan) for cells overexpressing WT integrin β4 or β4R1281W and seeded on laminin-coated glass coverslips; scale bar is 10 μm. Zoomed insert shows total integrin β4 (red) and GFP (cyan) stainings; scale bar is 5 μm. **e** Integrin β4 basal intensity (Normalized to 1.5 kPa WT condition) for cells overexpressing WT integrin β4 or β4R1281W (n = 39/28, 32/33, 44/35 for β4/β4R1281W and increasing rigidity; mean of 3 independent experiments). The effect of rigidity (*P* = 0.0002) and β4 mutation (*P* = 0.0079) is significant (two-way ANOVA, Bonferroni’s correction for multiple comparison). **f** Coefficient of variation of keratin 8 signal at the basal layer of the cell periphery for cells overexpressing WT integrin β4 or β4R1281W (n = 39/27, 29/32, 30/35 for β4/β4R1281W and increasing rigidity; mean of 3 independent experiments). The effect of rigidity and β4 mutation is significant (*P* < 0.0001 two-way ANOVA, Bonferroni’s correction for multiple comparison). **g** Sample images Integrin β4 (red) and keratin 8 (cyan) at the basal layer of WT integrin β4 or β4R1281W overexpressing cells on 11 kPa PAA gels; scale bar is 20 μm. **h** Integrin β4 basal intensity for MCF10A cells seeded on 11 kPa laminin- or collagen I-coated PAA gels (unpaired two-tailed t-test, n = 58/54; mean of 3 independent experiments). **i** Coefficient of variation of keratin 8 signal at the basal layer of the cell periphery for MCF10A cells seeded on 11 kPa laminin- or collagen I-coated PAA gels (unpaired two-tailed t-test, n = 57/60 from 3 independent experiments). **j** Sample images of Integrin β4 and keratin 8 (basal layer) for MCF10A cells seeded on 11 kPa laminin- or collagen I-coated PAA gels; scale bar is 20 μm. **k** Tractions for WT integrin β4 or β4R1281W overexpressing cells (n = 30/41, 30/29, 30/24, 31/28, 34/29, 40/32 cells for β4/β4R1281W and increasing rigidity; mean of 3 independent experiments). The effect of rigidity is significant (*P* < 0.0001, two-way ANOVA). **l** Tractions for WT integrin β4 or β4R1281W overexpressing cells on 11 kPa PAA gels (two-tailed Mann-Whitney test n = 31/28 cells; mean of 3 independent experiments). **m** Corresponding traction maps of WT or R1281W integrin β4 overexpressing cells; scale bar is 10 μm. **n** n/c YAP ratio for cells overexpressing WT integrin β4 or β4R1281W on 1.5 kPa laminin-coated PAA gels (n = 6/6 colonies; mean of 3 independent experiments). The effect of colony position (*P* < 0.0001) and β4 mutation (*P* = 0.0059) is significant (two-way ANOVA, Bonferroni’s correction for multiple comparison). **o** Sample YAP images of WT integrin β4 or β4R1281W cell colonies on 1.5 kPa; scale bar is 50 μm. **p** n/c YAP ratio for cells overexpressing WT integrin β4 or β4R1281W on 11 kPa laminin-coated PAA gels (n = 6/6 colonies; mean of 3 independent experiments). The effect of colony position (*P* < 0.0001) and β4 mutation (*P* = 0.0031) is significant (two-way ANOVA, Bonferroni’s correction for multiple comparison). **q** Sample YAP images of WT integrin β4 or β4R1281W cell colonies on 11 kPa; scale bar is 50 μm. **r** n/c YAP ratio of WT integrin β4 or β4R1281W overexpressing cells on 11 kPa PAA gels coated with laminin-332 (unpaired two-tailed t-test, n = 88/71 cells; mean of 3 independent experiments). **s** Coefficient of variation of keratin 8 signal at the basal layer of the cell periphery for cells overexpressing WT integrin β4 or β4R1281W on 11 kPa PAA gels coated with laminin-332 (unpaired two-tailed t-test, n = 69/59 cells, mean of 3 independent experiments). **t** Normalized keratin 8 signal at the basal layer of the cell periphery for WT integrin β4 or β4R1281W overexpressing cells on 11 kPa PAA gels coated with laminin-332 (unpaired two-tailed t-test, n = 69/59 cells; mean of 3 independent experiments). **u** Sample images of YAP and keratin 8 of WT integrin β4 or β4R1281W overexpressing cells on 11 kPa PAA gels coated with laminin-332; scale bar is 20 μm. Error bars represent mean ± s.e.m. Source data

Fiji software (ImageJ version 2.0.0/1.53g) was used to perform the image analysis, unless specified otherwise^63^. The length of p-Pax FAs was assessed, as described previously^64^, using the maximum projection of confocal images, by measuring the length of bright FAs on the edge of single cells and averaging the length of ten FAs per cell. The YAP *n*/*c* ratios were calculated, as described previously^3^, using the average projections of confocal images and by dividing the intensity on a nuclear region and a region with equal size in the cytosol immediately adjacent to the nuclear region on correcting for the background in cell-free zones. Background correction could not be applied for tissue sections, spheroids and colonies as cell-free zones were either absent or very distant. The corresponding Hoechst staining image and fluorescent staining signals were used to delimit the nuclear versus cytosolic regions. For Extended Data Figs. 2 and 3h–j, epifluorescence images were also used instead of confocal stacks. Due to the large number of conditions, each experimental repeat of FA and YAP measurements (Fig. 2a,b,d,e) included the control condition, but could not include all the conditions at the same time. To account for this, the values were corrected for each stiffness by the fold difference between the control value of the experiment, and the average value of all the control samples. Quantifications of the integrin β4 signal was carried out at the basal level of cells and normalized for each experiment to the 1.5 kPa levels (Extended Data Fig. 4e) and to the laminin levels (Extended Data Fig. 4h). Quantification of the coefficient of variation of the keratin 8 signal was carried out by measuring the standard deviation of the signal in three areas around the cell periphery marked by the actin signal at the basal surface of the single cells and averaged for each cell. The standard deviation was then normalized to the corrected mean intensity of the fluorescent signal. Keratin 8 intensity (Extended Data Fig. 4a) and cells labelled with lamin A/C (Extended Data Fig. 8b) were quantified on the sum projections of single cells or nuclei and were carried out using Fiji software. The sum H3K27m3 signal was quantified using Imaris.9 software (Oxford Instruments) on the three-dimensional segmentation of the cell nuclei. For each experiment, the sum (integrated density) signal was normalized to the average signal of β4 of the corresponding experiment to account for experimental variations.

### Western blots

Western blots were implemented following standard procedures. Briefly, the cells were lysed using RIPA buffer. Following denaturation, lysates were loaded on 4–20% PAA gels (Bio-Rad) and transferred onto a nitrocellulose membrane (Whatman, GE Healthcare Life Sciences). After blocking, the membranes were incubated with primary antibody overnight at 4 °C and with the horseradish-peroxidase-conjugated secondary (1:5,000) (Merck Millipore) antibody for 2 h at room temperature. ECL Western Blotting Substrate (Pierce, Thermo Fisher) was used to detect horseradish peroxidase and the bands were visualized with the ImageQuant LAS 4000 imaging system (GE Healthcare Life Sciences). The intensity of the bands was analysed using ImageJ software.

Antibodies used are as follows: mouse anti-lamin A/C (E1) 1:1,000 (Santa Cruz, category no. sc376248), mouse anti-integrin β4 (M126) 1:1,000 (Abcam, category no. ab29042), rabbit anti-GAPDH (D16H11) XP 1:1,000 (Cell Signaling, category no. 5174), mouse anti-GAPDH (6C5) 1:3,000 (Santa Cruz, category no. sc-3223), mouse anti-nesprin-3 antibody (Nsp3) 1:500 (Abcam, category no. ab123031), rabbit phospho-myosin light chain 2 (Thr18/Ser19) 1:500 (Cell Signaling, category no. 3674), rabbit anti-cytokeratin 8 (EP1628Y) 1:2,000 (Abcam, category no. ab53280).

Secondary horseradish-peroxidase-conjugated antibodies: goat anti-rabbit (Millipore, category no. AP132P) and donkey anti-mouse (Jackson Immunoresearch, category no. 715-035-151).

### Preparation, procedure and quantification of stretching experiments

The stretching experiments were carried out using a stretching device coupled to an upright Nikon ECLIPSE Ni-U microscope, as described before^3,40^. Briefly, stretchable membranes were prepared by mixing PDMS base and crosslinker at a 10:1 ratio, spinning the mixture for 1 min at 500 r.p.m. and finally cured overnight at 65 °C. Once cured, the PDMS membranes were placed on stretching devices and coated with 10 μg ml^–1^ laminin overnight at 4 °C. The cells were then seeded on the membranes and stretching experiments were carried out 4–8 h post-seeding, in an upright microscope (Nikon ECLIPSE Ni-U). Calibration of the system was done to adjust the vacuum applied to obtain 5% stretching of the PDMS surface. During stretching, the cells were kept in a CO_2_-independent medium (Thermo Fisher, category no. 18045088) containing 1:100,000 Hoechst 33258 staining dye and 10 μg ml^–1^ of rutin (Sigma R5143) to prevent photobleaching^65^ and treated with CellTracker (Invitrogen, category no. C34565). Membranes were subjected to equiaxial stretching on the application of vacuum and the images were acquired before and during stretching with a water-immersion ×60 objective (NA = 1.0). Changes in cell and nuclear strain were calculated by tracing the cell and nuclear perimeter using fluorescence signal and/or bright-field images and Hoechst signal, respectively, before and during stretching. A value of 0.1 has been assigned to negative-strain values.

### AFM experiments and quantification

Stiffness of cell nucleus and cytoplasm was measured with a NanoWizard 4 AFM (JPK) instrument mounted on top of a Nikon Ti ECLIPSE microscope^62^. The spring constant of the cantilevers was calibrated by thermal tuning using the simple harmonic oscillator model. The Hertz model was fitted to the approach curves to obtain the stiffness value using the JPKSPM Data Processing software (version 6.1.79). The cells were seeded on laminin-111-coated coverslips and a force curve on top of the nucleus and cytoplasm was acquired for each of the cells. The cells were kept at 37 °C using a BioCell (JPK) and maintained in a CO_2_-independent medium (Thermo Fisher, category no. 18045088).

### Optical tweezers experiments and quantification

Optical tweezers experiments were performed using a SENSOCELL (IMPETUX) device incorporated in a Ti ECLIPSE Nikon microscope, using a ×60 objective (Plan Apo; NA, 1.2; water-immersion type). Then, 1 μm carboxylate beads (01-02-103, Micromer) were coated with biotinylated laminin (LMN03, Cytoskeleton) or FN7-10 (a four-domain segment of fibronectin responsible for cell binding and containing the RGD and PHSRN motifs^66^) and biotinylated bovine serum albumin at a ratio of 1:10. The cells were seeded on #1.5 coverslips (Menzel-Gläser) previously coated with laminin. During the experiment, the cells were kept in a serum-free CO_2_-independent medium containing 1.0% penicillin–streptomycin (Gibco), 1.5% 1 M HEPES pH 7.5 (Sigma) and 2.0% l-glutamine (Gibco). Beads were flowed into the medium and were put into contact with cells on trapping them. A series of six oscillatory cycles of 12 s each and with an amplitude of 0.35 μm and frequency of 1 Hz were performed in an interval of 10 s for bead repositioning. The complex shear modulus G* was measured for each oscillation cycle using the microrheology routine of LightACE (1.6.2), the control software of the optical tweezers instrument. For the computation of G*, the force was determined by means of the calibration-free momentum method^67^ and the particle position was obtained using the measured stiffness of the trap.

### Cell monolayer experiments

Cells were seeded on 1.5 or 11.0 kPa gels of 18 mm diameter prepared, as described before, on MatTek dishes. On functionalization, 6 × 9 mm^2^ magnetic PDMS gaskets were placed on top and the plates were attached to a magnetic holder. Then, 1.2 × 10^5^ cells were seeded in the gasket and the non-attached cells were washed after 5 h. The cells were kept overnight in a serum-free medium before the removal of the magnetic gasket. For antibody-blocking experiments, a constant concentration (10 μg ml^–1^) of blocking antibodies or IgG control was maintained throughout the course of the experiment.

### Micropatterning

Circular (30 μm diameter) patterns were generated using the PRIMO micropatterning platform (Alvéole) on the surface of 12 kPa PDMS substrates, made as described elsewhere^68^. Briefly, PDMS CY 52-276 A and B (DOWSIL) were mixed at a 9:10 ratio on ice. The solution was then used to coat 35 mm MatTek dishes and cured at 65 °C overnight. The patterns were generated as per the manufacturer’s instruction. Beads were attached on the surface of the PDMS using an APTES-ethanol solution (5% v/v) (Sigma-Aldrich) to identify the surface of the gel. Gels were then passivated with a two-step incubation involving poly-l-lysine (Sigma-Aldrich, P2636) and PEG-SVA (Laysan Bio, MPEG-SVA-5000). Before micropatterning, the gels were covered with (*p*-benzoylbenzyl) trimethylammonium chloride (BOC Sciences) that allows ultraviolet-light-induced PEG degradation. The gels were then incubated with a 10 μg ml^–1^ laminin solution (1:1 rhodamine-labelled) (LMN01-A, Cytoskeleton) to non-labelled laminin (L2020, Sigma-Aldrich) solution.

### Traction force microscopy

Traction force experiments were performed, as described before^62,69^. Briefly, the cells were seeded on PAA gels of different rigidities, fabricated as described above. Traction force experiments were carried out using multidimensional acquisition routines on an automated inverted microscope (Nikon ECLIPSE Ti) equipped with thermal, CO_2_ and humidity control using MetaMorph (NIS Elements) imaging software. Fluorescent images of the beads and phase contrast images of the cells were acquired every 10 min during the experiment. Local gel deformation between any experimental time points and a reference image obtained after cell trypsinization were computed with a home-made PIV software implemented in MATLAB (MathWorks, version R2020b). Traction forces were computed using Fourier traction microscopy with a finite gel thickness^70^ and averaged for each cell.

### Actin and keratin retrograde flow experimental design and quantification

Cells were transfected with Lifeact-GFP and keratin 18-mCherry and seeded on PRIMO micropatterned PDMS, as described previously. Images were taken every 4 s using a Nikon TiE inverted microscope with a spinning-disc confocal unit (CSU-WD, Yokogawa) and a Zyla scientific complementary metal–oxide–semiconductor camera (Andor) controlled by µManager^71,72^ using a ×60 objective (Plan Apo; NA, 1.2; water-immersion type). The local velocity fields of the actin and keratin fluorescence signals were measured by comparing each frame and its previous time point with a custom-made PIV software in MATLAB. A mask of each cell was drawn with respect to the F-actin signal in ImageJ^73^. A radial coordinate, centred in the mask centroid, was assigned to each PIV data point, and normalized by the local radius of the cell-mask contour. Likewise, the local velocity fields were decomposed into their radial and tangential components. The distributions of the total and radial velocities inside each cell were then binned into equal-sized intervals of the normalized radial coordinate. The average total and radial velocity for each radial bin was then calculated.

### Cell transfection

Non-viral cell transfection was carried out using Lipofectamine 3000 Transfection Reagent (Invitrogen) following the manufacturer’s instructions. For the DN-KASH1 experiments, FACS selection was carried out on transfection. EGFP-nesprin-1-KASH or mCherry-nesprin-1-KASH and EGFP empty vector control and Lifeact-GFP were described previously^3,64,74^, pcDNA3.1-mCherry was a gift from D. Bartel (Addgene plasmid no. 128744; http://n2t.net/addgene:128744; RRID:Addgene_128744) (ref. ^75^) and mCherry-keratin 17 was a gift from M. Davidson (Addgene plasmid no. 55065; http://n2t.net/addgene:55065; RRID:Addgene_55065). siRNA transfection was performed using Lipofectamine RNAiMAX Transfection Reagent (Invitrogen), following the manufacturer’s instructions. Integrin β4 siRNA (category no. M-008011-02-0005) with NTC control (category no. D-001206-13-50) and nesprin-3 siRNA (category no. LQ-016637-12-0002) with NTC control (category no. D-001220-01-05) were purchased from Dharmacon. Retroviral particles for the generation of stable integrin β4-GFP and β4R1281W-GFP (LZRS-IRES-zeo plasmids were a gift from A. Sonnenberg^21^) lines were generated in HEK293T cells expressing retroviral packaging plasmids (gift from N. Montserrat) and transfected using Lipofectamine 3000 Transfection Reagent (Invitrogen).

### Blocking antibody and drug treatment experiments

Antibody-blocking experiments were performed by incubating the cells with control mouse IgG1, kappa monoclonal (MOPC-21) (ab18437, Abcam), anti-integrin β4 antibody clone ASC-8 (MAB2059Z), anti-integrin α3 clone P1B5 (MAB1952Z), anti-integrin β1 antibody clone P5D2 (MAB1959Z) (Merck Life Science, S.L.) and anti-integrin α6 antibody clone GoH3 (ab105669) (Abcam), for 20 min at room temperature before cell seeding and maintained in the same concentration (10 μg ml^–1^) of blocking antibodies throughout the duration of the experiment.

Drug treatment experiments were carried out by incubating cells with 25 μM blebbistatin, 50 μM CK-666 or the highest corresponding concentration of DMSO (Sigma-Aldrich) for 2 h on cell attachment and spreading on 11 kPa PAA gels coated with laminin (L2020, Sigma-Aldrich).

### Actin anisotropy quantification

Actin anisotropy was quantified in the maximum projection images from confocal stacks labelled with phalloidin. The anisotropy quantification was implemented using the ImageJ FibrilTool plug-in^76^.

### Keratin distribution quantification

Keratin and actin distributions were quantified on the average projections of keratin 8 and phalloidin actin of cells cultured on 30 μm patterns with a custom-made MATLAB code (version R2020b). As described above, a mask of each cell was drawn by thresholding for the actin signal using Fiji software^73^ and a normalized radial coordinate was assigned to each point of the image. An intensity profile was calculated by binning the normalized intensity values into equal-sized intervals of the normalized radial coordinate and averaging the values of intensity for each bin. Then, the profiles of intensity with respect to radial distance were normalized by the integral of the curve.

### Spheroid formation

Spheroids of MCF10A β4 and MCF10A β4R1281W cells were prepared in parallel. Briefly, AggreWell 400 dishes (STEMCELL Technologies, 34424) were pretreated with an anti-adherence solution (STEMCELL Technologies, 07010). Next, a single-cell suspension was prepared by adding 1× trypsin-EDTA (Thermo Fisher, 25200056). The cells were counted, diluted and seeded onto the AggreWell plate, according to the manufacturer’s protocol, to generate 1,000 cells per spheroid. The plate was carefully transferred to the incubator and the spheroids were allowed to form overnight.

### Alginate–Matrigel hydrogel preparation

Sodium alginate of an average molecular weight of 138 kDa (FMC Biopolymer) was used to prepare alginate–Matrigel interpenetrating networks of different stiffnesses, as described previously^77^. Briefly, the day before the experiment, alginate was reconstituted in DMEM/F12 (Gibco, 31331093). From this step forward, all the solutions were kept on ice. Next, an alginate–Matrigel mixture was prepared into intermediate concentrations of 1.25% and 5.4 mg ml^–1^, respectively (volumes must be adjusted based on the Matrigel batch concentration).

For each hydrogel, two Luer-Lok syringes were prepared (on ice). One syringe was filled with the alginate–Matrigel intermediate mixture, with sufficient volumes for a final concentration of 1% alginate and 4.4 mg ml^–1^ Matrigel. The second syringe containing the medium, spheroids (formed of MCF10A β4 or MCF10A β4R1281W) and calcium sulphate (final concentrations, 24.5 mM (stiff gels) and 8.9 mM (soft gels)). Next, both syringes were attached with a female–female Luer-Lok connector, taking care not to introduce bubbles or air into the mixture. The contents of the two syringes were rapidly mixed and the alginate gel was immediately deposited on top of a sterile plate and transferred to the incubator.

### Spheroid area quantification

Bright-field images of at least 50 spheroids per condition were taken every 24 h (from day 0 to day 5 of the experiments) with a ×4 objective in an EVOS M5000 microscope. The perimeter of each individual spheroid was manually drawn, and the enclosed area was measured using ImageJ.

### Nuclear shape quantification

Three-dimensional segmentation and shape characterization of Hoechst-33258-labelled nuclei was implemented using Imaris software (Oxford Instruments) via surface segmentation. Parameters used are as follows: sphericity, defined as $\varPsi =\frac{{\uppi }^{\frac{1}{3}}{\left(6{V}_{{\rm{p}}}\right)}^{\frac{2}{3}}}{{A}_{{\rm{p}}}}$, where *V*_p_ is the volume of the particle and *A*_p_ is the surface area of the particle; ellipsoid prolate, defined as ${e}_{{{\rm{prolate}}}}=\frac{{2\alpha }^{2}}{{\alpha }^{2}+{b}^{2}}\left(1-\frac{{\alpha }^{2}+{b}^{2}}{{2c}^{2}}\right)$; ellipsoid oblate, defined as ${e}_{{{\rm{oblate}}}}=\frac{{2b}^{2}}{{b}^{2}+{c}^{2}}\left(1-\frac{{2a}^{2}}{{b}^{2}+{c}^{2}}\right)$, where *a*, *b* and *c* are the lengths of the three semi-axes determining the shape of an ellipsoid (https://www.bitplane.com/download/manuals/ReferenceManual9_2_0.pdf).

Nuclear shapes of spheroids and tissue sections were calculated by drawing the perimeter of nuclei in a confocal image slide using Fiji.

### Immunofluorescence on patient tissue samples and quantification

Whole breast tissue sections of 25 invasive ductal carcinoma of no specific type and 6 mixed invasive ductal and lobular carcinoma, diagnosed as grades 1–3, were approved by the Tissue Science Committee of the University Medical Center Utrecht. Immunofluorescence was done on 4 µm whole breast tissue sections. Tissue sections of formalin-fixed paraffin-embedded invasive ductal carcinoma of no specific type were deparaffinized, followed by antigen retrieval by boiling in Tris-EDTA buffer for 20 min. Sections were washed with 1× PBS and blocked with 1% bovine serum albumin in PBS for 30 min before antibody and DAPI incubations. The slides were mounted in ProLong Diamond Antifade (Thermo Fisher P36961) and imaged after a 24 h drying period.

Antibodies used were as follows: mouse anti-laminin α-1 (CL3087) (Invitrogen, category no. MA5-31381), rabbit anti-YAP (D8H1X) XP (Cell Signaling, category no. 14074) and rat anti-cytokeratin 8 (Developmental Studies Hybridoma Bank, TROMA-I). Image acquisition was performed in a Nikon TiE inverted microscope with a spinning-disc confocal unit (CSU-WD, Yokogawa) and a Zyla scientific complementary metal–oxide–semiconductor camera (Andor), using a ×40 objective (Plan Fluor; NA, 0.75; dry). Quantification was performed in confocal slides of 31 different tumours. For each tumour sample, three images of areas defined by tumour boundaries were analysed. For each image, an average of laminin-α1 and keratin 8 intensity within the tumour boundaries was calculated, and average *n*/*c* YAP ratios were obtained by dividing the intensity on a nuclear region and a region with equal size in the cytosol immediately adjacent to the nuclear region. Averaged nuclear shapes within the tumour boundaries were also quantified by drawing the borders of the nuclei. Note that the nuclear shapes could only be quantified in two rather than three dimensions in this case.

### Statistical analysis

**Extended Data Fig. 5:**
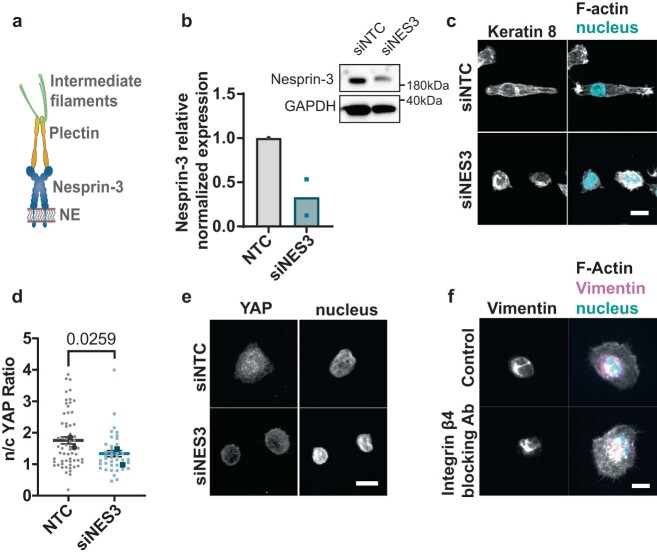
Characterization of the role of intermediate filament (IF)-nuclear interaction. **a** Schematic representation of the connection of nesprin-3 to intermediate filaments through plectin; NE = nuclear envelope. **b** Western blot for cells transfected with a control non-targeting siRNA (siNTC) or nesprin-3 siRNA (siNES3) and quantification (n = 2 independent experiments). **c** Keratin 8 and Phalloidin (F-actin) stainings for control and Nesprin-3 KD MCF10A cells on 11 kPa laminin-coated PAA gels; scale bar is 10 μm. **d** Quantification of YAP n/c ratios for non-targeting (siNTC) or nesprin-3 siRNA (siNES3) transfected MCF10A cells on 11 kPa laminin-coated PAA gels (unpaired two-tailed t-test, n = 60/39 control/siRNA transfected cells respectively; mean of 2 independent experiments). **e** Sample YAP stainings for siNTC or siNES3 transfected cells on 11 kPa laminin-coated PAA gels; scale bar is 10 μm. **f** Vimentin stainings for control (IgG) or integrin β4 blocking antibody treated cells on 11 kPa laminin-coated PAA gels; scale bar is 10 μm. Representative examples of n = 17/20 cells from 2 independent experiments. Error bars represent mean ± s.e.m. Source data

**Extended Data Fig. 6:**
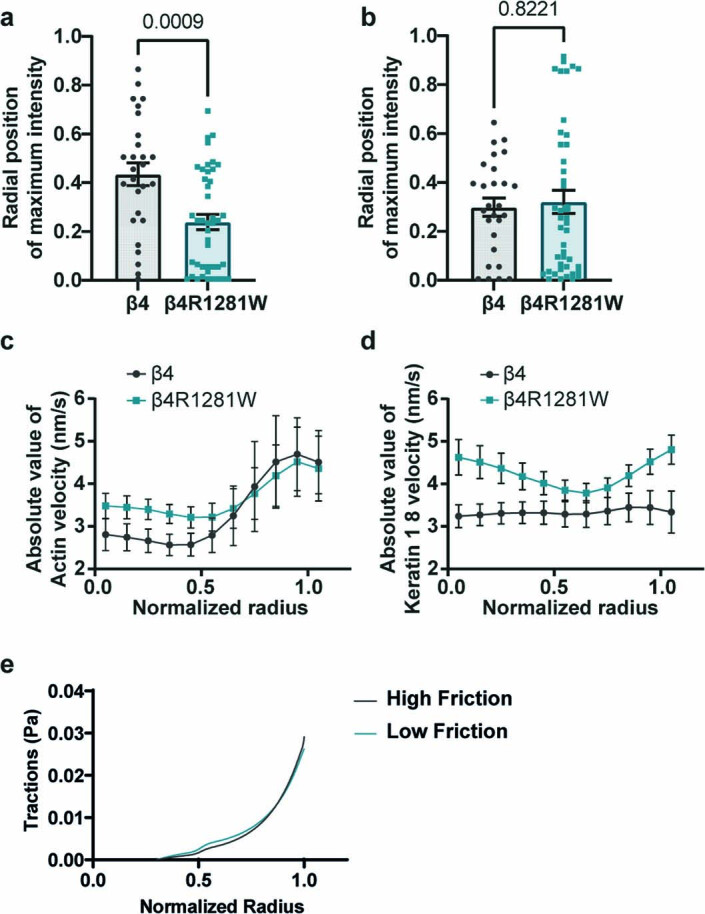
Further effects of integrin β4-keratin dissociation on cytoskeletal organisation and dynamics. **a** Plots showing the radial position of maximum keratin 8 signal for β4 or β4R1281W overexpressing cells seeded on 12 kPa laminin-patterned PDMS substrates. Radial position = 1, cell periphery, Radial position = 0, cell centre (two-tailed Mann-Whitney test, *n* = 27/43 cells for β4 and β4R1281W). **b** Plots showing the radial position of maximum actin signal for β4 or β4R1281W overexpressing cells seeded on 12 kPa laminin-patterned PDMS substrates (two-tailed Mann-Whitney test, *n* = 27/43 cells for β4 and β4R1281W). **c** Experimental quantifications of absolute actin retrograde flows along the cell radius for β4 or β4R1281W overexpressing cells seeded on 12 kPa laminin-patterned PDMS substrates (cell periphery R = 1 and centre R = 0). The combined effect of β4 mutation and radial actin velocities is not significant (*P* = 0.6927, two-way repeated measures ANOVA, *n* = 12/12 cells for β4 and β4R1281W). **d** Experimental quantifications of absolute keratin-18 retrograde flows along the cell radius for β4 or β4R1281W overexpressing cells seeded on 12 kPa laminin-patterned PDMS substrates. The combined effect of β4 mutation and radial keratin velocities is significant (*P* = 0.0073, two-way repeated measures ANOVA, *n* = 12/12 cells for β4 and β4R1281W). **e** Model prediction for radial tractions for high/low keratin-substrate friction (*η*_*0*_^*IF*^ of 8 versus 2 kPa s/µm^2^). Error bars represent mean ± s.e.m.

**Extended Data Fig. 7:**
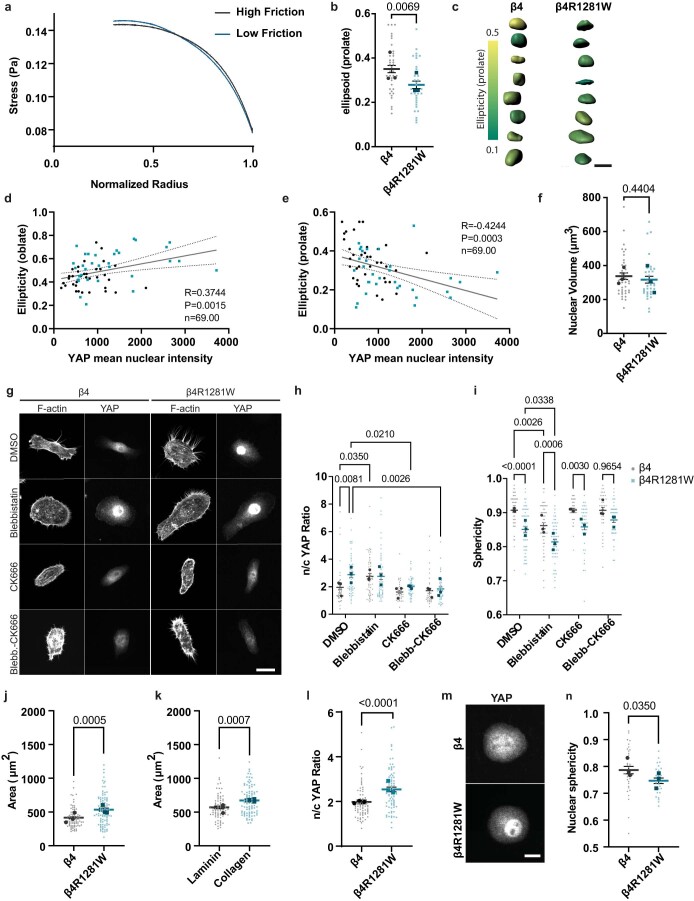
Further characterization of nuclear shape. **a** Model prediction for radial cell stress for high/low keratin-substrate friction (*η*_*0*_^*IF*^ of 8 versus 2 kPa s/µm^2^). Stress applied to the nucleus is the point corresponding to radius = 0.33. **b** Nuclear ellipticity (prolate) measurements of cells seeded on 11 kPa laminin-coated PAA gels (two-tailed unpaired t-test, n = 37/32 for β4/β4R1281W; mean of 3 independent experiments). **c** 3D segmentation and ellipticity (prolate) colour-coded nuclei for integrin β4 (left) and β4R1281W (right) overexpressing cells; scale bar is 10 μm. **d** Correlation between nuclear YAP intensity and ellipticity for both β4 (black circles) and β4R1281W (blue squares) integrin expressing cells (R is Spearman’s correlation coefficient). **e** Correlation between nuclear YAP intensity and ellipticity (prolate) for both β4 (black circles) and β4R1281W (blue squares) integrin expressing cells (R is Spearman’s correlation coefficient). **f** Nuclear volume measurements of cells seeded on 11 kPa laminin-coated PAA gels (two-tailed unpaired t-test, n = 37/32 for β4/β4R1281W; mean of 3 independent experiments). **g** Phalloidin (F-actin) and YAP stainings for integrin β4 and β4R1281W overexpressing cells seeded on 11 kPA PAA gels coated with laminin and treated with control DMSO, 25 μM Blebbistatin, 50 μM CK-666 or Blebbistatin + CK-666; Scale bar is 20 μm. **h** n/c YAP ratio quantification of integrin β4 and β4R1281W overexpressing cells seeded on 11 kPA PAA gels coated with laminin (n = 47/53, 54/52, 37/45, 35/42 for integrin β4 and β4R1281W overexpressing cells treated with DMSO, 25 μM Blebbistatin, 50 μM CK-666 or 25 μM Blebbistatin + 50 μM CK-666, respectively; mean of 3 independent experiments). The effect of both β4 mutation (*P* = 0.0079) and treatment (*P* < 0.0001) is significant (two-way ANOVA, Bonferroni correction for multiple comparisons). **i** Nuclear sphericity quantifications of integrin β4 and β4R1281W overexpressing cells seeded on 11 kPA PAA gels coated with laminin (n = 47/53, 54/52, 37/45, 35/42 for integrin β4 and β4R1281W transfected cells treated with DMSO, 25 μM Blebbistatin, 50 μM CK-666 or 25 μM Blebbistatin + 50 μM CK-666, respectively; mean of 3 independent experiments). The effect of both β4 mutation and treatment is significant. (*P* < 0.0001, two-way ANOVA, Bonferroni correction for multiple comparisons). **j** Area of WT integrin β4 or β4R1281W overexpressing cells on 11 kPa laminin-coated PAA gels (unpaired two-tailed t-test, n = 85/93; mean of 3 independent experiments). **k** Area of MCF10A cells on 11 kPa laminin- or collagen I-coated PAA gels (unpaired two-tailed t-test, n = 76/91; mean of 3 independent experiments). **l** n/c YAP ratio of WT integrin β4 or β4R1281W overexpressing cells on laminin- micropatterned 12 kPa PDMS substrates (unpaired two-tailed t-test, n = 77/80; mean of 3 independent experiments). **m** Sample YAP stainings of micropatterned cells; scale bar is 10 μm. **n** Nuclear sphericity of WT integrin β4 or β4R1281W overexpressing cells on laminin-micropatterned 12 kPa PDMS substrates (unpaired two-tailed t-test, n = 36/27, mean of 3 independent experiments). Error bars represent mean ± s.e.m.

**Extended Data Fig. 8:**
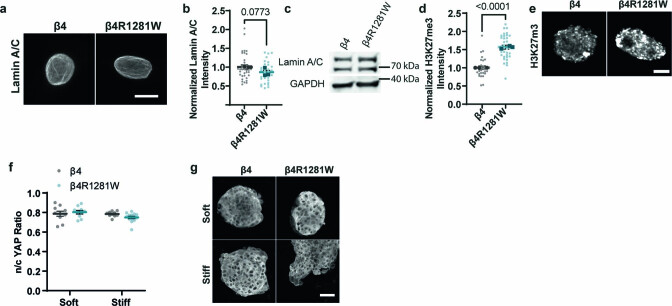
Lamin A/C, H3K27me3 and nuclear YAP levels during 3D invasion quantification of integrin β4 or β4R1281W expressing cells. **a** Lamin A/C stainings; scale bar is 10 μm. **b** Quantification of normalized (to β4 cells) intensity values (two-tailed unpaired t-test, n = 35/29 for β4/β4R1281W; mean of 3 independent experiments). **c** Western blot for Lamin A/C for integrin β4 and β4R1281W overexpressing cells (n = 2). **d** Quantification of H3K27me3 normalized intensity values (two-tailed unpaired t-test, n = 33/35 for β4/β4R1281W; mean of 3 independent experiments). **e** Nuclei stained for H3K27me3 stainings; scale bar is 5 μm. **f** Average n/c YAP ratios of WT integrin β4 or β4R1281W cell spheroids embedded in soft or stiff alginate-Matrigel matrices (n = 10/10, 10/14 spheroids for for soft and stiff β4/β4R1281W values; mean of 3 independent experiments, *P* > 0.05, two-way ANOVA). **g** Sample YAP images of WT integrin β4 or β4R1281W cell spheroids; scale bar is 50 μm. Error bars represent mean ± s.e.m. Source data

Statistical analyses were performed using GraphPad Prism software (version 9). Statistical significance was determined by the specific tests indicated in the corresponding figure legends. Non-parametric tests were performed when neither original nor log-10-transformed datasets were normally distributed. All the experiments presented here were repeated in at least three independent experiments, except for fibronectin and collagen I conditions (Extended Data Fig. 2 (*n* = 2), Extended Data Fig. 5b,d (*n* = 2) and Extended Data Fig. 8c (*n* = 2)). In the dot plots throughout the manuscript, experimental repeat averages are illustrated with darker and bigger points.

### Reporting summary

Further information on research design is available in the Nature Portfolio Reporting Summary linked to this article.

## Online content

Any methods, additional references, Nature Portfolio reporting summaries, source data, extended data, supplementary information, acknowledgements, peer review information; details of author contributions and competing interests; and statements of data and code availability are available at 10.1038/s41563-023-01657-3.

### Supplementary information

Supplementary Information Supplementary Notes 1–5, Fig. 1 and Tables 1 and 2.

Reporting Summary

Supplementary Video 1 **Actin and keratin velocities for WT-integrin-β4-expressing cells**. Spinning-disc confocal time-lapse imaging of a cell co-transfected with Lifeact-GFP and keratin 18-mCherry, seeded on a laminin-coated circular pattern. Scale bar, 20 μm.

Supplementary Video 2 **Actin and keratin velocities for integrin-β4R1281W-expressing cells**. Spinning-disc confocal time-lapse imaging of a cell co-transfected with Lifeact-GFP and keratin 18-mCherry, seeded on a laminin-coated circular pattern. Scale bar, 20 μm.

### Source data

Source data Fig. 2 and Extended Data Figs. 3, 4, 5 and 8 Unprocessed western blots.

## Data Availability

The data that support the findings of this study are available in the Article, Extended Data and Supplementary Information. The other relevant data are available from the corresponding authors upon request and also available at 10.34810/data747. Source data are provided with this paper.

## Code availability

The codes used in this study are available from the corresponding authors on request. The computational model of the actomyosin and keratin networks is available via GitLab at https://gitlab.com/PSaez83/actinkeratincell2022.
