# Supplementary material for: The laminin–keratin link shields the nucleus from mechanical deformation and signalling
Source: Nat Mater. 2023 Sep 14;22(11):1409–20. doi: 10.1038/s41563-023-01657-3 (PMC10627833; doi:10.1038/s41563-023-01657-3)
Supplement: Supplementary file 1 — Supplementary Notes 1–5, Fig. 1 and Tables 1 and 2. [file 41563_2023_1657_MOESM1_ESM.pdf]

nature materials

Article

https://doi.org/10.1038/s41563-023-01657-3

The laminin–keratin link shields the nucleus from mechanical deformation and signalling

In the format provided by the authors and unedited

Supplementary notes

Supplementary note 1. Controls on the differential role of laminin versus collagen or fibronectin.

To assess the differential effect of laminin in cell mechanosensing, we carried out different controls. First, we checked that coating densities in our PAA gels of laminin, collagen I, or fibronectin were comparable (Extended data fig. 1a-c). Further, decreasing or increasing laminin coating by an order of magnitude had no effect on cell response (Extended data fig. 1d,e). Thus, impaired mechanosensing on laminin was not due to deficient coating. Next, we evaluated ECM deposition by cells during the 6-hour period allowed between cell seeding and measurements. Cells seeded on all substrate types secreted laminin, which showed a clear staining in the basal plane of cells. In contrast, cells did not secrete collagen I or fibronectin, as these stainings only showed a weak non-specific cytosolic signal that was not localised to the basal plane of cell-matrix contact (Extended data fig. 1f). Thus, cells seeded on collagen I or fibronectin matrices are also exposed to laminin to a certain degree. Still, this laminin is not sufficient to override collagen I or fibronectin responses, which were markedly different. This implies that mechanosensing responses should be restored for gels coated with a mixture of laminin and increasing amounts of collagen I. This was indeed confirmed (Extended data fig. 1g-i). Finally, and further validating the applicability of these results to the breast context, we found similar responses for human breast myoepithelial cells. Those cells exhibited an increase in n/c YAP ratios and FA length with rigidity (from 0.5 to 30 kPa) on collagen I or fibronectin coated substrates (Extended data fig. 2). In contrast, cells seeded on laminin had similar n/c YAP ratios when seeded on 0.5 and 30 kPa gels, and a small increase in FA length (Extended data fig. 2).

Supplementary note 2. Direct transmission of force to the nucleus via keratin.

To explore how laminin regulates nuclear mechanoresponses, we first hypothesised that the keratin cytoskeleton could directly affect force transmission to the nucleus, which is known to trigger YAP nuclear localisation[1]. To assess this, we knocked down nesprin-3, which connects the keratin cytoskeleton to the nuclear lamina[2] (Extended data fig. 5a,b). Nesprin-3 depletion strongly impacted cell phenotype, leading to cell rounding, perinuclear collapse of the actin and keratin 8 cytoskeleton, as well as low YAP n/c ratios (Extended data fig. 5c-e). This effect was opposite to that induced by blocking α6β4 or its connection to keratins. Thus, a direct link between keratins and the nucleus via nesprin-3 does not explain our results, although it highlights that the interaction between the keratin cytoskeleton and the nucleus is important in maintaining cellular integrity. Of note, this result discards a direct role not only of keratin but also other intermediate filaments (IFs) expressed by these cells, such as vimentin, which can also bind to the nucleus via nesprin-3. Further, vimentin did not seem to strongly interact with hemidesmosomes, as it was distributed exclusively around the nucleus, with no appreciable difference between control and integrin β4 blocking antibody treated cells (Extended data fig. 5f).

Supplementary note 3. Computational model of actomyosin and keratin networks

Geometric model and model considerations

To understand the mechanics of the actomyosin and keratin networks of the cell and the mechanical interaction between these two networks and between the networks and the substrate, we developed a mathematical model of the cell composite cytoskeleton, which we solved numerically using the finite element method. We consider a 1D axisymmetric domain of length 2/3R modelling the region comprised between the nucleus edge and the cell edge for a stationary and radially symmetric cell (Supplementary Fig. 1a). Thus, flows are only considered in the radial direction. Our model is composed of an F-actin network that is actively pulled by myosin motors, as modelled previously[3–5], and a viscoelastic network of intermediate filaments (IF) that is dragged by the F-actin flow. We show a sketch of the model in Supplementary Fig. 1b,c. We thus consider a cellular domain along coordinate x and given by (R/3, R), where r = R/3 represents the nuclear boundary of the composite network and r = R represents the cell front (Supplementary Fig. 1a,c). We assume that each network has a well-defined hydrodynamic velocity field, $v^a(r)$ for actin and $v^{IF}(r)$ for IF, and can sustain a mechanical stress, $\sigma^a(r)$ and $\sigma^{IF}(r)$ respectively. Furthermore, these networks interact with their surroundings and between each other frictionally, modeling both unspecific mechanisms and the net mechanical effect of transient binding between specific ligands. Each network possesses distinct constitutive laws as detailed next.

Mechanics of the F-Actin network

To model the mechanical behaviour of the F-Actin network and, ultimately, compute the velocity of the F-actin network, v^a, we describe the balance of linear momentum neglecting inertial forces along with boundary conditions as described previously[3–5] (using polar coordinates r, θ):

$$\frac{\partial \sigma^a_{rr}}{\partial r} + \frac{\sigma^a_{rr}}{r} - \frac{\sigma^a_{\theta\theta}}{r} = \eta^a v^a + \eta(v^a - v^{IF}) \quad \text{in } (R/3, R)$$

$$v^a(r = R/3) = 0 \qquad (1)$$

$$v^a(r = R) = v^a{}_f$$

where we considered $v^a{}_f = -1.5\text{x}10^{-3}$ µm/s for the actin velocity at the cell edge, modelling polymerisation

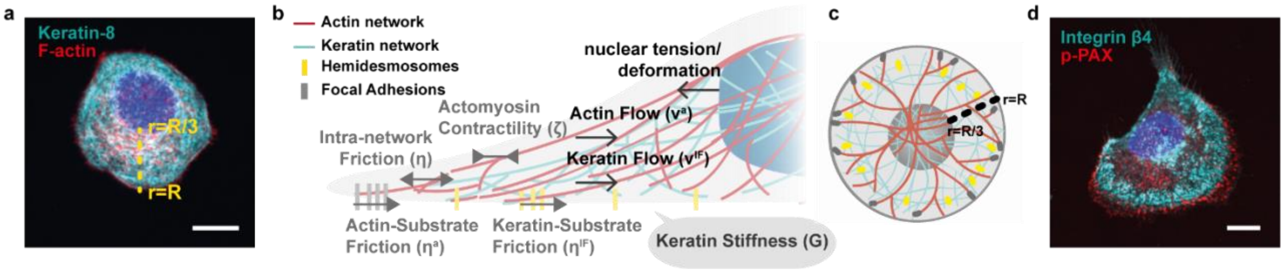

Supplementary Figure 1: (a) Example of a micropatterned cell stained for actin and keratin 8. In the model, the cell is considered as a circular axisymmetric system, with radial dimension r ranging from 0 (cell centre) to R (cell edge). The nucleus is placed in a range from r=0 to r=R/3. Scale bar is 10 µm. (b) Intracellular structure of the model (shown as a vertical cut for illustration purposes only). The network of actin fibres is shown in red and keratin fibres in cyan. Actomyosin contractility leads to actin retrograde flow, resisted by actin-substrate and actin-keratin friction. Due to applied force through actomyosin, keratin also undergoes retrograde flow, resisted by keratin-substrate friction. The keratin-substrate link mediated by integrin α6β4 is modelled by regulating keratin-substrate friction, and keratin cytoskeletal stiffness. The extranuclear networks can impose compressive and tensional forces on the nucleus through actin-nuclear links, leading to nuclear deformation. Model inputs in grey, model outputs in black. (c) Top view of model components, reproducing the axisymmetric geometry considered by the model. The model considers the cytoskeletal region outside of the nucleus, ranging from r=R/3 to r=R. (d) Focal adhesions, as depicted by phospho-Paxillin staining (pPAX in red) and hemidesmosomes (integrin β4 adhesions in cyan), are spatially accumulated in the periphery of the cell (as considered by the model); scale bar is 10 µm.

at the lamellipodium, and zero actin velocity at the nucleus, in agreement with experimental results (Fig. 4j). The second term in the right-hand side corresponds to frictional forces due to the relative motion between actin and the IF network controlled by the constant friction coefficient η. The first term models the frictional forces resulting from the sliding of the actin network relative to the extracellular matrix (ECM) with friction coefficient η^a. We modulate friction between actin and the ECM, η^a, in space following a sigmoidal distribution:

$$\eta^a = \eta^a{}_0[1+\frac{1}{(1+\exp(-a(r-rFA))}] \qquad (2)$$

Which adopts a baseline value $\eta^a{}_0$ close to the nucleus, and increases to nearly $2\eta^a{}_0$ at the cell front, with $\eta^a(r_{FA})=0.5\eta^a{}_0$ for $r_{FA} = 0.8R$. α is a constant that sets the slope of the sigmoidal function. This space-dependent function accounts for the fact that focal adhesions mainly form at the periphery of the cell[6,7], as we also show in Supplementary Fig. 1d. This parameter is important because we model mutant cells to have a weaker interaction with the laminin ECM, by reducing $\eta^a{}_0$.
We consider the constitutive relation for the internal stresses of the F-actin network as:

$$\sigma^a_{rr} = \mu^a \partial_r v^a + \zeta\rho^a\rho^m \text{ and } \sigma^a_{\theta\theta} = \mu^a v^a/r + \zeta\rho^a\rho^m \qquad (3)$$

where the first term considers the viscosity of the actin network and the second term models the contractile forces of the myosin motors. μ^a is the shear viscosity, ζ the active contraction exerted by the contractile myosin motors. ρ^m and ρ^a are the density of the myosin motors and the actin network specified below.

Mechanics of the IF network

To model the mechanics of the IF network and obtain its velocity field v^{IF}, we define an analogous equation for balance of linear momentum[8] along with boundary conditions

$$\frac{\partial\sigma^{IF}_{rr}}{\partial r} + \frac{\sigma^{IF}_{rr}}{r} - \frac{\sigma^{IF}_{\theta\theta}}{r} = \eta^{IF} v^{IF} - \eta(v^a - v^{IF}) \qquad \text{in (R/3,R)}$$

$$\sigma^{IF}(r=R/3) = 0 \qquad (4)$$

$$v^{IF}(r=R) = 0$$

where we impose zero velocity at the nucleus[9], which is also consistent with results (Fig. 4k). We impose that the network is traction-free at the cell front.

As for the actin network, the first term in the right-hand side represents the friction between the IF network and the ECM, which we assume to be proportional to the IF network velocity with friction constant η^{IF}. Following the same argument as for the interaction of the F-actin network with the ECM, we assume the same space dependency of η^{IF}. Note, however, that this spatial dependence had a minor effect on the results. The second term is the friction resulting from inter-network relative motion.
We model the constitutive response of the IF network as a viscoelastic solid obeying:

$$\sigma^{IF}_{rr} = \mu^{IF}\partial_r v^{IF} - G(\lambda - 1) \text{ and } \sigma^{IF}_{\theta\theta} = \mu^{IF} v^{IF}/r - G(\lambda - 1) \qquad (5)$$

where the first term accounts for the viscosity of the IF network with shear viscosity μ^{IF} and the second term is elastic stress, where G is the bulk elastic modulus and λ is the volumetric stretch ratio of the network. Based on previous data, we consider G to be proportional to the density of the IF network as $G = G_0\rho^{IF}$ [10], where G_0 is the elastic modulus at unit normalised density. The elastic

modulus of the keratin network is also assumed to increase as the crosslinking of keratin network increases[11,12]. Through hemidesmosomes, keratin network attachment to the substrate will increase network crosslinking[13]. To introduce this aspect in the simplest possible way, we vary G_0 proportionally to substrate friction, which also depends on hemidesmosomes, following $G_0 = G_0^* \eta^{IF}{}_0 / \eta^{IF*}{}_0$. We choose $G_0^* = 0.5 kPa$ at a reference friction $\eta^{IF*}{}_0 = 5\ kPa \cdot s/\mu m^2$ such that the value of G_0 in the WT and β4R1281W cases fits the respective bulk elastic moduli of the experimental data (Fig. 4o,p). Of note, experimental bulk moduli capture contributions of all cytoskeletal networks and not only IFs. In our model, we assign these values to the IF network for simplicity due to its major role. Importantly, the differences between the WT and mutant conditions (which are the main focus of the model) are indeed caused specifically by changes in the IF network.

The relation between network stretch and velocity is given by the kinematic relation $\partial_t \lambda + \partial_r(v^{IF}\lambda) = 0$ [8]. However, to model the fact that elastic stresses dissipate over time, e.g. as a result of turnover, or equivalently that the volumetric strain λ-tends to one as the network is rebuilt with a rate constant τ^{λ}, we consider the following previously considered advection-reaction equation for the evolution of λ [8]

$$\partial_t \lambda + \partial_r(f^{\lambda}) + f^{\lambda}/r = -\tau^{\lambda}(\lambda - 1) \quad \text{in } (R/3,R),\ t > 0, \qquad (6)$$

where $f^{\lambda} = v^{IF}\lambda$.

Model of F-actin, myosin and IF density

To describe the distribution in time- and space of the intracellular F-actin, myosin and IF densities, we consider three transport advection-diffusion reaction equations. We model the effective transport of the actin network density, ρ^a, as:

$$\partial_t \rho^a + \partial_r f^a + f^a / r = k^a{}_p - k^a{}_d \rho^a \quad \text{in } (R/3,R),\ t > 0, \qquad (7)$$

where $f^a = v^a \rho^a - \nu^a \partial_r \rho^a, \nu^a$ is the diffusion constant, and $k^a{}_p$ *and* $k^a{}_d$ are the polymerization and depolymerization rates of the network, respectively. We impose $k^a{}_p = k^a{}_d$ to normalize the density to one. We impose homogeneous natural boundary conditions at both sides of the domain.
Similarly, we model the effective transport of the IF network density, ρ^{IF}, as:

$$\partial_t \rho^{IF} + \partial_r f^{IF} + f^{IF}/r = k^{IF}{}_p - k^{IF} \rho^{IF}{}_d \quad \text{in } (R/3,R),\ t > 0 \qquad (8)$$

Where $f^{IF} = v^{IF}\rho^{IF} - \nu^{IF}\partial_r \rho^{IF}, \nu^{IF}$ is the diffusion constant, and $k^{IF}{}_p$ *and* $k^{IF}{}_d$ are the polymerization and and depolymerization rates of the IF network. We impose $k^{IF}{}_p = k^{IF}{}_d$ to normalize the density to one at steady state. We impose again zero flux boundary conditions at each cell end to describe that the IF network cannot enter or leave the cell membrane in the front of the cell and to reflect symmetry boundary conditions at the nuclear side.
Finally, we model the effective transport of myosin motor density bound to the F-actin network, ρ^m, as a transient advection-diffusion problem[14]:

$$\partial_t \rho^m + \partial_r f^m + f^m/r = 0 \quad \text{in } (R/3,R),\ t > 0, \qquad (9)$$

Where $f^m = v^a \rho^m - \nu^m \partial_r \rho^m$. This equation reflects the assumption of a much faster attachment rate of motors to the F-actin network than the detached rate into the unbound cytosolic form. Effectively, bound myosin motors are advected with velocity v^a [5,14,15] and diffuse with diffusion constant ν^m. Again, we impose zero flux boundary conditions at both ends.

Model of nuclear deformation and sphericity

The composite network model described above allows us to compute the total stress at the nucleus by evaluating $\sigma^a + \sigma^{IF}$ at r = R/3. We obtain a total stress of 0.120kPa and 0.119kPa for the control and mutant cases, respectively. We model the nucleus as a linear element so that nuclear strain e is given by $\sigma^{IF} + \sigma^a = G^N e$, where G^N is the nuclear elastic modulus taken from Fig. 4Q. Given e, and hence the in-plane linear stretch $\alpha = 1+e$, and assuming equibiaxial strain and nuclear incompressibility, we compute the out-of-plane linear stretch as $1/\alpha^2 = 1/(1+e)^2$. Thus, an initially spherical nucleus of radius R ends up as an oblate ellipsoid with in-plane semiaxes R(1+e) and out-of-plane semiaxis $R/(1+e)^2$, which provides an estimate for nuclear shape and allows us to compute sphericity. We obtain a sphericity of 0,97 and 0,79 for control and mutant cells.

Modelling intracellular network dynamics in WT cells

To verify our model, we first simulate the dynamics of the intracellular networks in the WT cells and validate the results against the experimental data. Our computational model includes a number of model parameters that do not change as we change conditions of this study. These model parameters are presented in Supplementary Table 1. Note that many of these model parameters are obtained from the literature, although we allowed for slight variations to better fit experimental results. Regarding the diffusion parameters of the actin and keratin networks, we assumed that they do not diffuse and are transported mainly because of convection. Therefore, we introduced small diffusive terms to stabilize the numerical solution of the equations. For the turnover rates of the system, we assumed that the attachment of free myosin is fast, so that the effective transport equation for the attached myosin motors is given as in Eq. 9**.** We assumed that actin turnover is 2 orders of magnitude faster than that of the keratin network.

Supplementary Table 1: Material parameters of the computational model of the acto-myosin and IF network. All references were used to obtained magnitudes of the corresponding parameters. * indicates that the values were fitted in this work.

**Meaning and units**	**Actin**			**IF**		
	**Symbol**	**Value**	**Ref.**	**Symbol**	**Value**	**Ref.**
Viscosity [kPa·s]	μ^a	30	[14,16]	μ^{IF}	10	*
Friction with substrate [kPa·s/µm]	η^a	8	[14,16,17]	η_0^{IF}	8	*
Contractility [kPa]	ζ	0.13	[5,16]		-	-
Stiffnes [kPa]		-	-	G_0^*	0.5	*
Polymerization/depolymerization rates	k^a_p / k^a_d	0.1/0.1	[18,19]	k^{IF}_p / k^{IF}_d	0.001/0.001	*
Diffusion constant [µm²/s]	ν^a	0.001	*	ν^{IF}	0.001	*
friction between actin and IF networks[kPa·s/µm]	η	4	*	η	4	*

Our results for WT cells show an accumulation of IFs close to the nucleus while F-actin density is more uniformly distributed along the cell, both in agreement with experimental results (Fig. 4e,f). Actin flow is powered by the active pulling of myosin motors that has to balance the friction with the ECM and with the IFs (Supplementary Fig. 1b). The actin retrograde flow decays quickly from 1.5 nm/s at the cell front to zero at the nucleus (main text Fig. 4g). The IF network flow does not have intrinsic activity in our model and is dragged by the motion of the actin network and experiences the friction with the ECM. The velocity of IFs follows a similar decay from the cell periphery to the nucleus, but it is 1 order of magnitude slower than actin flow (main text Fig. 4h). As a result of these dynamics, the IF network accumulates close to the nucleus and imposes a viscous stress to the cell nucleus.

Results also show that the actin network progressively goes from a tension state of 0.0784 kPa, close to the cell membrane, to 0.1438 kPa at the nucleus. The IF network is elastically in compression in the entire cell domain, except for a small region close to the cell membrane where it is stretched. At the nuclear region, IFs exert a compressive stress of 0.3Pa. Therefore, total tension applied to the cell nucleus is 0.1435 kPa. The frictional forces on the ECM, resulting from actin and IF flows, are high at the cell periphery, as observed experimentally. It is also important to note that the frictional forces applied by the actin network through FAs, are higher (1 order of magnitude) than the frictional forces that the IF network applies on the ECM. Again, this is in close agreement with the experimental observations (no change in traction forces, Extended data fig. 4k-m).

Sensitivity analysis of the model parameters

Parameters in Supplementary Table 1 were either taken from literature or adjusted during simulations to fit the different experimental data. We perform a sensitivity analysis for those parameters. The sensitivity S was calculated according to the following expression[8]:

$$S = d \log \Psi \,/\, d\log p$$

Where p are values of the different parameters, and Ψ is the value of nuclear sphericity. S values can be interpreted as the fold change in Ψ induced by a fold change in the parameter value. To calculate S values, parameters were varied ¼ and 4-fold from the optimal fitted values. Then, Ψ was plotted against p in a log-log scale, and S was taken as the slope of a linear fit to the plot.

Supplementary Table 2: Sensitivity analysis of the model parameters. The default parameters used to fit the model results to the control and mutant conditions are in Supplementary Table 1. For those parameters, sensitivity values report how the nuclear sphericity at the nucleus depends on changes in the parameter values. Positive/negative values indicate that the threshold rigidity increases/decreases as the parameter increases.

Parameter	Range	Sensitivity
μ^a	7,5-120	-10^{-3}
μ^{IF}	2,5-40	$8\mathrm{x}10^{-5}$
G_0^*	0,05-0,8	$4\mathrm{x}10^{-4}$
ζ	0,075-1,5	-0.019
η	1,0-16,0	0,02
η^a	4,0-16,6	$9\mathrm{x}10^{-4}$
η_0^{IF}	0,5-32	0, 399
ν^{a}	10^{-4}-10^{-2}	$-2.9\mathrm{x}10^{-5}$
ν^{IF}	10^{-4}-10^{-2}	$-5\mathrm{x}10^{-6}$
$K^a{}_p$ / $k^a{}_d$	0,005-0,05	$3\mathrm{x}10^{-4}$
$k^{IF}{}_p$ / $k^{IF}{}_d$	0,005-0,05	$-9\mathrm{x}10^{-6}$

Modelling intracellular network dynamics in β4R1281W mutant cells

We then simulate the network dynamics in β4R1281W mutant cells. We assume that the main change in the system is a reduction in the friction of IFs with the substrate, and therefore we lower η_0^{IF} from 8 to 2 kPa s/μm^2. Mutant cells also have decreased keratin-substrate cross-linking and thereby decreased elasticity, which, as explained above, is modelled by introducing a linear relation between $\eta^{IF}{}_0$ and G_0.

Our results show that the mutant condition leads to negligible effects in the density and velocity of the actomyosin cytoskeleton and in the corresponding traction forces on the substrate. A small increase in nuclear stress (1.6%) occurs because reduced IF-substrate friction decreases the stiffness of the IF network and, therefore, reduces the compressive forces applied to the nucleus. However, the main effects on nuclear strain are not explained by this small increase in stress but by a decrease in the elasticity (stiffness) of the IF network around the cell nucleus. IFs are crosslinked to the nucleus and contribute to its mechanical properties[2,11,12], and thus according to the model, the overall stiffness of the nucleus and its surrounding cytoskeleton should decrease, as verified experimentally (Fig. 4o,p). Overall, the combined effects of increased stress and decreased stiffness lead to an increase in nuclear strain for the mutant cells, promoting nuclear mechanotransduction.

Supplementary note 4. Cytoskeletal velocities and tractions, and comparison with the model.

Overall cytoskeletal velocities (including non-radial) for cells seeded on circular micropatterns were higher for the mutant condition, especially for the keratin network, indicating a looser network (Extended data fig. 6c,d and Supplementary Videos 1 and 2). Interestingly, the model does not predict large effects of the β4R1281W integrin mutant on traction forces, because intracellular forces are transmitted to the substrate through the actin cytoskeleton (Extended data fig. 6e). This is consistent with our finding that blocking or interfering with integrin β4-keratin binding did not affect traction forces (Fig. 2g-i and Extended data fig. 4k-m). Thus, the link between keratin and the substrate through integrin β4 can withstand actomyosin mediated contractility, affecting the organisation of the keratin network and decreasing its retrograde flow.

Supplementary note 5. Relationship between YAP levels, nuclear shapes, and actin-mediated forces.

To complete the mechanical characterisation of our system, we carried out measurements of both nuclear shape and YAP nuclear localisation after inhibiting myosin contractility (with blebbistatin), actin polymerisation (by blocking specifically the Arp2/3 complex using the CK666 inhibitor[20]), or both. Interestingly and as previously described[21], blebbistatin-treated cells had high nuclear YAP levels, were more spread and had deformed nuclei with low sphericities (Extended data fig. 7g-i). This suggests that in the absence of myosin contractility, unopposed actin polymerisation spreads and flattens cells and nuclei. Indeed, perinuclear Arp2/3-driven actin polymerisation has been previously shown to drive nuclear deformation[22]. Confirming this, both nuclear flattening and YAP nuclear localisation were reverted by blocking actin polymerisation (Extended data fig. 7g-i). Further supporting the control of YAP by nuclear mechanics, there was a high correlation between YAP levels and nuclear shape (Fig. 5k). As the only exception to this rule and suggesting a saturation of YAP n/c ratios, blebbistatin-treated integrin β4R1281W expressing cells decreased nuclear sphericity with respect to WT integrin β4 expressing cells without further increasing YAP levels (Fig. 5k and Extended data fig. 7g-i). Finally, treatment with both inhibitors diminished both nuclear YAP levels and resulted in similar levels of sphericity for both β4 and β4R1281W integrin-expressing cells (Fig. 5k and Extended data fig. 7g-i).

This correlation between cell spreading and nuclear shape has been previously reported[23], and is, in fact, consistent with our proposed hypothesis (although not explicitly considered by our theoretical model). Indeed, reduced keratin anchoring to the substrate should not only affect retrograde flows as discussed above but also reduce resistance to actin spreading. Consistently, cells expressing mutant integrin β4 spread more than cells expressing WT β4 (Extended data fig. 7j), and cells seeded on collagen I (where actin- rather than keratin-binding integrins dominate) spread more than cells seeded on laminin (Extended data fig. 7k). However, if the substrate-keratin interaction mechanically shields the nucleus, nuclear shapes and nuclear YAP levels should also be affected even if cells spreading is not altered. Indeed, similar changes in both nuclear sphericity and nuclear YAP levels were observed between WT and mutant integrin β4 cells even when cells were plated on equally sized micropatterns (Extended data fig. 7l-n). Thus, we show that keratin cytoskeleton stiffening can prevent actin-mediated nuclear deformation.

Supplementary References:

1. Elosegui-Artola, A. *et al.* Force Triggers YAP Nuclear Entry by Regulating Transport across Nuclear Pores. *Cell* **171**, 1397-1410.e14 (2017).

2. Wilhelmsen, K. *et al.* Nesprin-3, a novel outer nuclear membrane protein, associates with the cytoskeletal linker protein plectin. *J. Cell Biol.* **171**, 799–810 (2005).

3. Prost, J., Jülicher, F. & Joanny, J. F. Active gel physics. *Nature Physics* vol. 11 111–117 (2015).

4. Recho, P., Putelat, T. & Truskinovsky, L. Contraction-driven cell motility. *Phys. Rev. Lett.* **111**, 108102 (2013).

5. Rubinstein, B., Fournier, M. F., Jacobson, K., Verkhovsky, A. B. & Mogilner, A. Actin-myosin viscoelastic flow in the keratocyte lamellipod. *Biophys. J.* **97**, 1853–1863 (2009).

6. Gardel, M. L., Schneider, I. C., Aratyn-Schaus, Y. & Waterman, C. M. Mechanical integration of actin and adhesion dynamics in cell migration. *Annual Review of Cell and Developmental Biology* vol. 26 315–333 (2010).

7. Parsons, J. T., Horwitz, A. R. & Schwartz, M. A. Cell adhesion: Integrating cytoskeletal dynamics and cellular tension. *Nature Reviews Molecular Cell Biology* vol. 11 633–643 (2010).

8. Lewis, O. L., Guy, R. D. & Allard, J. F. Actin-myosin spatial patterns from a simplified isotropic viscoelastic model. *Biophys. J.* **107**, 863–870 (2014).

9. Pora, A. *et al.* Regulation of keratin network dynamics by the mechanical properties of the environment in migrating cells. *Sci. Rep.* **10**, 1–17 (2020).

10. Elbalasy, I., Mollenkopf, P., Tutmarc, C., Herrmann, H. & Schnauß, J. Keratins determine network stress responsiveness in reconstituted actin–keratin filament systems. *Soft Matter* (2021) doi:10.1039/d0sm02261f.

11. Kim, J. S., Lee, C. H., Su, B. Y. & Coulombe, P. A. Mathematical modeling of the impact of actin and keratin filaments on keratinocyte cell spreading. *Biophys. J.* **103**, 1828–1838 (2012).

12. Sivaramakrishnan, S., DeGiulio, J. V., Lorand, L., Goldman, R. D. & Ridge, K. M. Micromechanical properties of keratin intermediate filament networks. *Proc. Natl. Acad. Sci. U. S. A.* **105**, 889–894 (2008).

13. Borradori, L. & Sonnenberg, A. Structure and function of hemidesmosomes: More than simple adhesion complexes. *J. Invest. Dermatol.* **112**, 411–418 (1999).

14. Putelat, T., Recho, P. & Truskinovsky, L. Mechanical stress as a regulator of cell motility. *Phys. Rev. E* **97**, 012410 (2018).

15. Hawkins, R. J. *et al.* Pushing off the walls: A mechanism of cell motility in confinement. *Phys. Rev. Lett.* **102**, (2009).

16. Larripa, K. & Mogilner, A. Transport of a 1D viscoelastic actin-myosin strip of gel as a model of a crawling cell. *Phys. A Stat. Mech. its Appl.* **372**, 113–123 (2006).

17. Bergert, M. *et al.* Force transmission during adhesion-independent migration. *Nat. Cell Biol.* **17**, 524–529 (2015).

18. Wilson, C. A. *et al.* Myosin II contributes to cell-scale actin network treadmilling through network disassembly. *Nature* **465**, 373–377 (2010).

19. Raz-Ben Aroush, D. *et al.* Actin Turnover in Lamellipodial Fragments. *Curr. Biol.* **27**, 2963-2973.e14 (2017).

20. Hetrick, B., Han, M. S., Helgeson, L. A. & Nolen, B. J. Small molecules CK-666 and CK-869 inhibit actin-related protein 2/3 complex by blocking an activating conformational change. *Chem. Biol.* **20**, 701–712 (2013).

21. Das, A., Fischer, R. S., Pan, D. & Waterman, C. M. YAP nuclear localization in the absence of cell-cell contact is mediated by a filamentous actin-dependent, Myosin IIand Phospho-YAP-independent pathway during extracellular matrix mechanosensing. *J. Biol. Chem.* **291**, 6096–6110 (2016).

22. Thiam, H. R. *et al.* Perinuclear Arp2/3-driven actin polymerization enables nuclear deformation to facilitate cell migration through complex environments. *Nat. Commun.* **7**, 1–14 (2016).

23. Khatau, S. B. *et al.* A perinuclear actin cap regulates nuclear shape. *Proc. Natl. Acad. Sci. U. S. A.* **106**, 19017–19022 (2009).
