# Supplementary material for: The laminin–keratin link shields the nucleus from mechanical deformation and signalling
Source: Nat Mater. 2023 Sep 14;22(11):1409–20. doi: 10.1038/s41563-023-01657-3 (PMC10627833; doi:10.1038/s41563-023-01657-3)
Supplement: Supplementary file 2 — Reporting Summary [file 41563_2023_1657_MOESM2_ESM.pdf]

nature portfolio

Corresponding author(s): Pere Roca-Cusachs, Zanetta Kechagia

Last updated by author(s): Jun 8, 2023

Reporting Summary

Nature Portfolio wishes to improve the reproducibility of the work that we publish. This form provides structure for consistency and transparency in reporting. For further information on Nature Portfolio policies, see our Editorial Policies and the Editorial Policy Checklist.

Statistics

For all statistical analyses, confirm that the following items are present in the figure legend, table legend, main text, or Methods section.

n/a	Confirmed	
☐	☒	The exact sample size (*n*) for each experimental group/condition, given as a discrete number and unit of measurement
☐	☒	A statement on whether measurements were taken from distinct samples or whether the same sample was measured repeatedly
☐	☒	The statistical test(s) used AND whether they are one- or two-sided<br>*Only common tests should be described solely by name; describe more complex techniques in the Methods section.*
☒	☐	A description of all covariates tested
☐	☒	A description of any assumptions or corrections, such as tests of normality and adjustment for multiple comparisons
☐	☒	A full description of the statistical parameters including central tendency (e.g. means) or other basic estimates (e.g. regression coefficient) AND variation (e.g. standard deviation) or associated estimates of uncertainty (e.g. confidence intervals)
☐	☒	For null hypothesis testing, the test statistic (e.g. *F*, *t*, *r*) with confidence intervals, effect sizes, degrees of freedom and *P* value noted<br>*Give P values as exact values whenever suitable.*
☒	☐	For Bayesian analysis, information on the choice of priors and Markov chain Monte Carlo settings
☒	☐	For hierarchical and complex designs, identification of the appropriate level for tests and full reporting of outcomes
☐	☒	Estimates of effect sizes (e.g. Cohen's *d*, Pearson's *r*), indicating how they were calculated

Our web collection on statistics for biologists contains articles on many of the points above.

Software and code

Policy information about availability of computer code

Data collection	Image acquisition software Metamorph (Version 7.7.10), Micromanager (version 1.4.22), ZEN (ZEISS, version 2.3 SP1 FP3 black). Optical tweezers data were generated using LightAce 1.6.2 Software. AFM data was acquired using the JPK software (JPK Data Processing Version 6.1.79).
Data analysis	Immunostaining images were analyzed with ImageJ (version 1.53g) and Imaris.9 (© Oxford Instruments). Data statistic tests were performed with Graphpad PRISM (version 9). LightACE 1.6.2 was used for rheological data processing. JPKSPM data processing software (JPK Data Processing Version 6.1.79) for AFM stiffness value processing. Actin anisotropy was analyzed using FibrilTool ImageJ plugin. Traction force microscopy data were generated using a custom particle imaging velocimetry (PIV) software implemented in Matlab (MathWorks Inc.) (version R2019b). Keratin distribution quantification was performed using a custom-made MATLAB code (version R2020b). Actin and keratin flows were quantified using a custom-made PIV software in MATLAB (version R2020b). The computational model of actomyosin and keratin networks was generated in MATLAB (version R2020b) and can be find at https://gitlab.com/PSaez83/actinkeratincell2022.git . Other custom-made codes are available upon request.

For manuscripts utilizing custom algorithms or software that are central to the research but not yet described in published literature, software must be made available to editors and reviewers. We strongly encourage code deposition in a community repository (e.g. GitHub). See the Nature Portfolio guidelines for submitting code & software for further information.

Data

Policy information about availability of data

All manuscripts must include a data availability statement. This statement should provide the following information, where applicable:

- Accession codes, unique identifiers, or web links for publicly available datasets
- A description of any restrictions on data availability
- For clinical datasets or third party data, please ensure that the statement adheres to our policy

Values used to generate the graphs of this manuscript are available at https://doi.org/10.34810/data747

Field-specific reporting

Please select the one below that is the best fit for your research. If you are not sure, read the appropriate sections before making your selection.

☒ Life sciences ☐ Behavioural & social sciences ☐ Ecological, evolutionary & environmental sciences

For a reference copy of the document with all sections, see nature.com/documents/nr-reporting-summary-flat.pdf

Life sciences study design

All studies must disclose on these points even when the disclosure is negative.

Sample size	Sample size was calculated based on previous experiments and pilot experiments.
Data exclusions	No data were excluded.
Replication	Data were replicated at least 3 times for most experiments and 2 times for the ones listed in the manuscript, obtaining similar results. The number for the repeats is stated for each data set.
Randomization	All allocations and measurements were random.
Blinding	2 people performed experiments and data analysis for integrin beta4 mutant versus control YAP nuclear ratios. No experiment was blinded, because the same investigator performed and analyzed the data. Analysis of TFM, AFM, optical tweezers, actin anisotropy, nuclear shapes, fluorescence intensity, actin-keratin distribution and flow experiments are not blinded since they cannot be influenced by the subjected judgment of the examiner.

Behavioural & social sciences study design

All studies must disclose on these points even when the disclosure is negative.

Study description	*Briefly describe the study type including whether data are quantitative, qualitative, or mixed-methods (e.g. qualitative cross-sectional, quantitative experimental, mixed-methods case study).*
Research sample	*State the research sample (e.g. Harvard university undergraduates, villagers in rural India) and provide relevant demographic information (e.g. age, sex) and indicate whether the sample is representative. Provide a rationale for the study sample chosen. For studies involving existing datasets, please describe the dataset and source.*
Sampling strategy	*Describe the sampling procedure (e.g. random, snowball, stratified, convenience). Describe the statistical methods that were used to predetermine sample size OR if no sample-size calculation was performed, describe how sample sizes were chosen and provide a rationale for why these sample sizes are sufficient. For qualitative data, please indicate whether data saturation was considered, and what criteria were used to decide that no further sampling was needed.*
Data collection	*Provide details about the data collection procedure, including the instruments or devices used to record the data (e.g. pen and paper, computer, eye tracker, video or audio equipment) whether anyone was present besides the participant(s) and the researcher, and whether the researcher was blind to experimental condition and/or the study hypothesis during data collection.*
Timing	*Indicate the start and stop dates of data collection. If there is a gap between collection periods, state the dates for each sample cohort.*
Data exclusions	*If no data were excluded from the analyses, state so OR if data were excluded, provide the exact number of exclusions and the rationale behind them, indicating whether exclusion criteria were pre-established.*
Non-participation	*State how many participants dropped out/declined participation and the reason(s) given OR provide response rate OR state that no participants dropped out/declined participation.*

Randomization	*If participants were not allocated into experimental groups, state so OR describe how participants were allocated to groups, and if allocation was not random, describe how covariates were controlled.*

Ecological, evolutionary & environmental sciences study design

All studies must disclose on these points even when the disclosure is negative.

Study description	*Briefly describe the study. For quantitative data include treatment factors and interactions, design structure (e.g. factorial, nested, hierarchical), nature and number of experimental units and replicates.*
Research sample	*Describe the research sample (e.g. a group of tagged Passer domesticus, all Stenocereus thurberi within Organ Pipe Cactus National Monument), and provide a rationale for the sample choice. When relevant, describe the organism taxa, source, sex, age range and any manipulations. State what population the sample is meant to represent when applicable. For studies involving existing datasets, describe the data and its source.*
Sampling strategy	*Note the sampling procedure. Describe the statistical methods that were used to predetermine sample size OR if no sample-size calculation was performed, describe how sample sizes were chosen and provide a rationale for why these sample sizes are sufficient.*
Data collection	*Describe the data collection procedure, including who recorded the data and how.*
Timing and spatial scale	*Indicate the start and stop dates of data collection, noting the frequency and periodicity of sampling and providing a rationale for these choices. If there is a gap between collection periods, state the dates for each sample cohort. Specify the spatial scale from which the data are taken*
Data exclusions	*If no data were excluded from the analyses, state so OR if data were excluded, describe the exclusions and the rationale behind them, indicating whether exclusion criteria were pre-established.*
Reproducibility	*Describe the measures taken to verify the reproducibility of experimental findings. For each experiment, note whether any attempts to repeat the experiment failed OR state that all attempts to repeat the experiment were successful.*
Randomization	*Describe how samples/organisms/participants were allocated into groups. If allocation was not random, describe how covariates were controlled. If this is not relevant to your study, explain why.*
Blinding	*Describe the extent of blinding used during data acquisition and analysis. If blinding was not possible, describe why OR explain why blinding was not relevant to your study.*

Did the study involve field work? ☐ Yes ☐ No

Field work, collection and transport

Field conditions	*Describe the study conditions for field work, providing relevant parameters (e.g. temperature, rainfall).*
Location	*State the location of the sampling or experiment, providing relevant parameters (e.g. latitude and longitude, elevation, water depth).*
Access & import/export	*Describe the efforts you have made to access habitats and to collect and import/export your samples in a responsible manner and in compliance with local, national and international laws, noting any permits that were obtained (give the name of the issuing authority, the date of issue, and any identifying information).*
Disturbance	*Describe any disturbance caused by the study and how it was minimized.*

Reporting for specific materials, systems and methods

We require information from authors about some types of materials, experimental systems and methods used in many studies. Here, indicate whether each material, system or method listed is relevant to your study. If you are not sure if a list item applies to your research, read the appropriate section before selecting a response.

Materials & experimental systems

n/a	Involved in the study	
☐	☒	Antibodies
☐	☒	Eukaryotic cell lines
☒	☐	Palaeontology and archaeology
☒	☐	Animals and other organisms
☐	☒	Human research participants
☒	☐	Clinical data
☒	☐	Dual use research of concern

Methods

n/a	Involved in the study	
☒	☐	ChIP-seq
☒	☐	Flow cytometry
☒	☐	MRI-based neuroimaging

Antibodies

Antibodies used

The primary antibodies used, and their respective dilutions for immunofluorescence are: Rabbit Phospho-Paxillin 1:100 (Tyr118) (Cell Signaling, Cat# 69363 and 2541s), RRID:AB_2174466), rabbit anti-YAP (D8H1X) XP® 1:100 (Cell Signaling, Cat# 14074, RRID:AB_2650491), mouse anti-YAP1 YAP1 (63.7) 1:100 (Santa Cruz, Cat# sc-101199, RRID:AB_1131430), rabbit anti-Cytokeratin 8 [EP1628Y] 1:200 (Abcam, Cat# ab53280, RRID:AB_869901), rabbit anti-plectin antiserum 1:400 (#46, gift from Gerhard Wiche), mouse anti-Integrin beta 4 [M126] 1:1000 (Abcam, Cat# ab29042, RRID:AB_870635), mouse Anti-Lamin A + Lamin C antibody [131C3] 1:200 (Abcam, Cat# ab8984, RRID:AB_306913), mouse anti-Lamin A/C (E1) 1:100 (Santa Cruz, Cat# sc376248, RRID:AB_10991536), rabbit anti-Tri-Methyl-Histone H3 (Lys27) (C36B11) 1:300 (Cell Signaling, Cat# 9733, RRID:AB_2616029), anti-Laminin α-1 (CL3087) (Invitrogene, Cat# MA5-31381, RRID:AB_2787018), rabbit anti-Laminin 1:200 (Abcam, Cat# ab11575, RRID:AB_298179), anti-Collagen I 1:200 (Millipore, Cat# AB755P, RRID:AB_11211912), anti-Fibronectin 1:200 (Sigma, Cat# F3648, RRID:AB_476976), rabbit anti-Vimentin 1:250 (Abcam, Cat# ab92547, RRID:AB_10562134), Rat anti-Cytokeratin 8 (Developmental Studies Hybridoma Bank, TROMA-I).
The secondary antibodies used are: mouse Alexa Fluor -488 (Cat# A-11029, RRID:AB_2534088), -555 (Cat# A-21424, RRID:AB_141780 and Cat# A-31570, RRID AB_2536180), -647 (Cat# A-21236, RRID:AB_2535805) and rabbit Alexa Fluor -488 (Cat# A-21206, RRID:AB_2535792), -555 (Cat# A-21429, RRID:AB_2535850), -647 (Cat# A-21245, RRID:AB_2535813) all at 1:300 concentration (ThermoFisher).

Antibodies used for western blot are: mouse anti-Lamin A/C (E1) 1:1000 (Santa Cruz, Cat# sc376248, RRID:AB_10991536), mouse anti-Integrin beta 4 [M126] 1:1000 (Abcam, Cat# ab29042, RRID:AB_870635), rabbit anti-GAPDH (D16H11) XP® 1:1000 (Cell Signaling, Cat# 5174, RRID:AB_10622025), mouse anti-GAPDH (6C5) 1:3000 (Santa Cruz, Cat# sc-32233, RRID:AB_627679), mouse Anti-Nesprin3 antibody [Nsp3] 1:500 (Abcam Cat# ab123031, RRID:AB_10975264), rabbit Phospho-Myosin Light Chain 2 (Thr18/Ser19) 1:500 (Cell Signaling, Cat# 3674, RRID:AB_2147464), rabbit anti-Cytokeratin 8 [EP1628Y] 1:2000 (Abcam, Cat# ab53280, RRID:AB_869901).Secondary HRP conjugated antibodies: Goat anti-Rabbit (Millipore, Cat# AP132P, RRID:AB_90264) and Donkey anti-Mouse (Jackson Immunoresearch, Cat# 715-035-151, RRID:AB_2340771)

Antibodies for function-blocking experiments at 10 µg/ml: Anti-Integrin ß4 Antibody clone ASC-8 (MAB2059Z, RRID:AB_94526), Anti-Integrin α3, clone P1B5 (Millipore, MAB1952Z, RRID:AB_11213492), Anti-Integrin β1 Antibody, clone P5D2 (Millipore, MAB1959Z, RRID:AB_94462) (Merck Life Science, S.L.), Anti-Integrin alpha 6 antibody, clone GoH3 (Abcam, ab105669, RRID:AB_10860152) and mouse IgG1, kappa monoclonal [MOPC-21] (Abcam, ab18437 (Abcam).

Validation

All antibodies used for this study have been validated by the respective manufacturers and by previous studies, including our own experiments. Our experiments used immunofluorescence staining to validate antibodies (localisation of antibody staining) and/or gene knockdown and overexpression experiments. All antibodies were purchased from commercial vendors.

The primary antibodies: Rabbit Phospho-Paxillin (Tyr118) ((https://www.cellsignal.com/products/primary-antibodies/phospho-paxillin-tyr118-e9u9f-rabbit-mab/69363) and 2541s (https://www.cellsignal.com/products/primary-antibodies/phospho-paxillin-tyr118-antibody/2541?site-search-type=Products&N=4294956287&Ntt=2541s&fromPage=plp&_requestid=2155208), rabbit anti-YAP (D8H1X) XP® (https://www.cellsignal.com/products/primary-antibodies/yap-d8h1x-xp-rabbit-mab/14074), mouse anti-YAP1 YAP1 (63.7) (https://www.scbt.com/p/yap-antibody-63-7), rabbit anti-Cytokeratin 8 [EP1628Y] (https://www.abcam.com/products/primary-antibodies/cytokeratin-8-antibody-ep1628y-cytoskeleton-marker-ab53280.html), rabbit anti-plectin antiserum (#46, described here: Andrä, K. et al. Plectin-isoform-specific rescue of hemidesmosomal defects in plectin (-/-) keratinocytes. J. Invest. Dermatol. 120, 189–197 (2003)), mouse anti-Integrin beta 4 [M126] (https://www.abcam.com/products/primary-antibodies/integrin-beta-4-antibody-m126-ab29042.html), mouse Anti-Lamin A + Lamin C antibody [131C3] (https://www.abcam.com/products/primary-antibodies/lamin-a--lamin-c-antibody-131c3-nuclear-envelope-marker-ab8984.html), mouse anti-Lamin A/C (E1) (https://www.scbt.com/p/lamin-a-c-antibody-e-1), rabbit anti-Tri-Methyl-Histone H3 (Lys27) (C36B11) (https://www.cellsignal.com/products/primary-antibodies/tri-methyl-histone-h3-lys27-c36b11-rabbit-mab/9733), anti-Laminin α-1 (CL3087) (https://www.thermofisher.com/antibody/product/Laminin-alpha-1-Antibody-clone-CL3087-Monoclonal/MA5-31381), rabbit anti-Laminin (https://www.abcam.com/products/primary-antibodies/laminin-antibody-ab11575.html), anti-Collagen I (https://www.merckmillipore.com/CH/de/product/Anti-Rat-Collagen-Type-I-Antibody,MM_NF-AB755P?ReferrerURL=https%3A%2F%2Fwww.google.com%2F), anti-Fibronectin (https://www.sigmaaldrich.com/CH/de/product/sigma/f3648), rabbit anti-Vimentin (https://www.abcam.com/products/primary-antibodies/vimentin-antibody-epr3776-cytoskeleton-marker-ab92547.html), Rat anti-Cytokeratin 8 (https://www.sigmaaldrich.com/ES/es/product/mm/mabt329m), Rabbit anti-GAPDH (D16H11) XP® (https://www.cellsignal.com/products/primary-antibodies/gapdh-d16h11-xp-rabbit-mab/5174), mouse anti-GAPDH (6C5) (https://www.scbt.com/p/gapdh-antibody-6c5), mouse Anti-Nesprin3 antibody [Nsp3] (https://www.abcam.com/products/primary-antibodies/nesprin3-antibody-nsp3-ab123031.html), rabbit Phospho-Myosin Light Chain 2 (Thr18/Ser19) (https://www.cellsignal.com/products/primary-antibodies/phospho-myosin-light-chain-2-thr18-ser19-antibody/3674), Anti-Integrin ß4 Antibody clone ASC-8 (https://www.sigmaaldrich.com/CH/de/product/mm/mab2059z), Anti-Integrin α3, clone P1B5 (https://www.sigmaaldrich.com/CH/de/product/mm/mab1952z), Anti-Integrin β1 Antibody, clone P5D2 (https://www.sigmaaldrich.com/CH/de/product/mm/mab1959), Anti-Integrin alpha 6 antibody, clone GoH3 (https://www.abcam.com/products/primary-antibodies/integrin-alpha-6-antibody-goh3-ab105669.html) mouse IgG1, kappa monoclonal [MOPC-21] (file:///Users/jk/Downloads/datasheet_18437.pdf).

The secondary antibodies: mouse Alexa Fluor -488 (https://www.thermofisher.com/antibody/product/Goat-anti-Mouse-IgG-H-L-Highly-Cross-Adsorbed-Secondary-Antibody-Polyclonal/A-11029), -555 (https://www.thermofisher.com/antibody/product/Goat-anti-Mouse-IgG-H-L-Highly-Cross-Adsorbed-Secondary-Antibody-Polyclonal/A-21424 and https://www.thermofisher.com/antibody/product/Donkey-anti-Mouse-IgG-H-L-Highly-Cross-Adsorbed-Secondary-Antibody-Polyclonal/A-31570), -647 (https://www.thermofisher.com/antibody/product/Goat-anti-Mouse-IgG-H-L-Highly-Cross-Adsorbed-Secondary-Antibody-Polyclonal/A-21236) and rabbit Alexa Fluor -488 (https://www.thermofisher.com/antibody/product/Donkey-anti-Rabbit-IgG-H-L-Highly-Cross-Adsorbed-Secondary-Antibody-Polyclonal/A-21206), -555 (https://www.thermofisher.com/antibody/product/Goat-anti-Rabbit-IgG-H-L-Highly-Cross-Adsorbed-Secondary-Antibody-Polyclonal/A-21429), -647 (https://www.thermofisher.com/antibody/product/Goat-anti-Rabbit-IgG-H-L-Highly-Cross-Adsorbed-Secondary-Antibody-Polyclonal/A-21245), HRP conjugated antibodies: Goat anti-Rabbit

(https://www.thermofisher.com/antibody/product/32260.html) and Donkey anti-Mouse (https://www.jacksonimmuno.com/catalog/products/715-035-151)

Eukaryotic cell lines

Policy information about cell lines

Cell line source(s)	Mammary epithelial cells (MCF 10A) were purchased from ATCC (Cat# CRL-10317). Immortalized myoepithelial cell line (1089) were obtained from J. Louise Jones, Barts Cancr Institute, and are described in Clin Cancer Res. 2014 Jan 15;20(2):344-57. doi: 10.1158/1078-0432.CCR-13-1504. HEK293T cells for retroviral production were a gift from Prof. N. Montserrat (Institute for Bioengineering of Catalonia).
Authentication	None of the cell lines used were authenticated.
Mycoplasma contamination	All cell lines were tested for Mycoplasma contamination regularly and were negative.
Commonly misidentified lines (See ICLAC register)	*Name any commonly misidentified cell lines used in the study and provide a rationale for their use.*

Palaeontology and Archaeology

Specimen provenance	*Provide provenance information for specimens and describe permits that were obtained for the work (including the name of the issuing authority, the date of issue, and any identifying information). Permits should encompass collection and, where applicable, export.*
Specimen deposition	*Indicate where the specimens have been deposited to permit free access by other researchers.*
Dating methods	*If new dates are provided, describe how they were obtained (e.g. collection, storage, sample pretreatment and measurement), where they were obtained (i.e. lab name), the calibration program and the protocol for quality assurance OR state that no new dates are provided.*

☐ Tick this box to confirm that the raw and calibrated dates are available in the paper or in Supplementary Information.

Ethics oversight	*Identify the organization(s) that approved or provided guidance on the study protocol, OR state that no ethical approval or guidance was required and explain why not.*

Note that full information on the approval of the study protocol must also be provided in the manuscript.

Animals and other organisms

Policy information about studies involving animals; ARRIVE guidelines recommended for reporting animal research

Laboratory animals	*For laboratory animals, report species, strain, sex and age OR state that the study did not involve laboratory animals.*
Wild animals	*Provide details on animals observed in or captured in the field; report species, sex and age where possible. Describe how animals were caught and transported and what happened to captive animals after the study (if killed, explain why and describe method; if released, say where and when) OR state that the study did not involve wild animals.*
Field-collected samples	*For laboratory work with field-collected samples, describe all relevant parameters such as housing, maintenance, temperature, photoperiod and end-of-experiment protocol OR state that the study did not involve samples collected from the field.*
Ethics oversight	*Identify the organization(s) that approved or provided guidance on the study protocol, OR state that no ethical approval or guidance was required and explain why not.*

Note that full information on the approval of the study protocol must also be provided in the manuscript.

Human research participants

Policy information about studies involving human research participants

Population characteristics	All patients had confirmed breast cancer (morphology/breast cancer biomarker subclassification stainings e.g. ER/PR/HER2 for invasive ductal carcinomas and aberrant or presence E-cadherin for invasive lobular carcinoma). The median age was 51 years old.
Recruitment	This study does not entail any clinical trials or specific patient recruitment. Samples were obtained through standard care. After diagnostics, the samples used in this study were considered leftover material and used pseudonymised for biomarker validation.
Ethics oversight	No study protocols were involved, the samples are not subjected to a designated study, and as such, do not need ethical approval. Informed consent forms to use left-over tissue for research purposes were signed and collected at the UMC Utrecht patients' treatment team. Ethical oversight is provided by the local bodies (TCBio: https://tcbio.umcutrecht.nl/en/)

Note that full information on the approval of the study protocol must also be provided in the manuscript.

Clinical data

Policy information about clinical studies

All manuscripts should comply with the ICMJE guidelines for publication of clinical research and a completed CONSORT checklist must be included with all submissions.

Clinical trial registration	*Provide the trial registration number from ClinicalTrials.gov or an equivalent agency.*
Study protocol	*Note where the full trial protocol can be accessed OR if not available, explain why.*
Data collection	*Describe the settings and locales of data collection, noting the time periods of recruitment and data collection.*
Outcomes	*Describe how you pre-defined primary and secondary outcome measures and how you assessed these measures.*

Dual use research of concern

Policy information about dual use research of concern

Hazards

Could the accidental, deliberate or reckless misuse of agents or technologies generated in the work, or the application of information presented in the manuscript, pose a threat to:

No	Yes	
☐	☐	Public health
☐	☐	National security
☐	☐	Crops and/or livestock
☐	☐	Ecosystems
☐	☐	Any other significant area

Experiments of concern

Does the work involve any of these experiments of concern:

No	Yes	
☐	☐	Demonstrate how to render a vaccine ineffective
☐	☐	Confer resistance to therapeutically useful antibiotics or antiviral agents
☐	☐	Enhance the virulence of a pathogen or render a nonpathogen virulent
☐	☐	Increase transmissibility of a pathogen
☐	☐	Alter the host range of a pathogen
☐	☐	Enable evasion of diagnostic/detection modalities
☐	☐	Enable the weaponization of a biological agent or toxin
☐	☐	Any other potentially harmful combination of experiments and agents
