# Supplementary material for: The laminin–keratin link shields the nucleus from mechanical deformation and signalling
Source: Nat Mater. 2023 Sep 14;22(11):1409–20. doi: 10.1038/s41563-023-01657-3 (PMC10627833; doi:10.1038/s41563-023-01657-3)

Fig. 2J

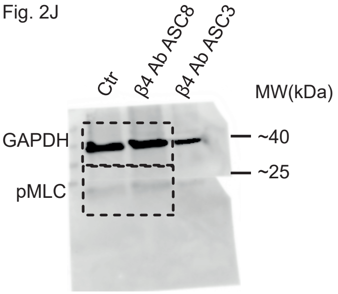

Extended data Fig. 3a

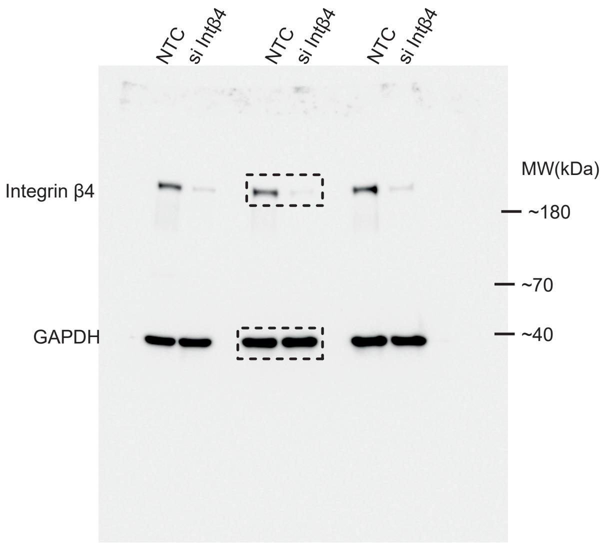

Extended data Fig. 3g

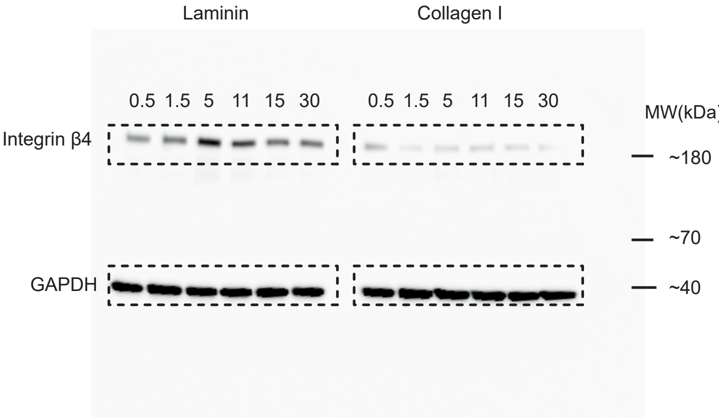

Extended data Fig. 5b

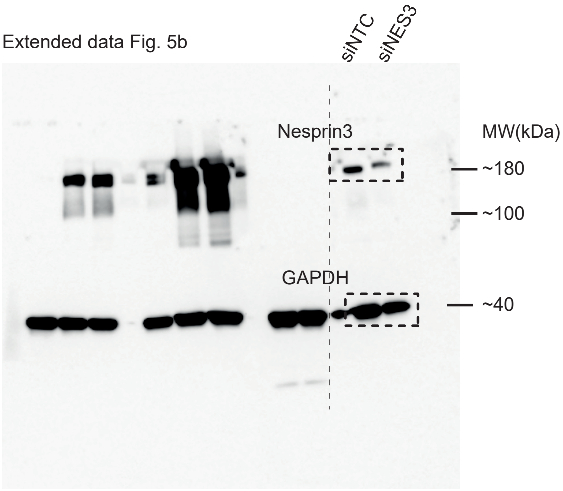

Extended data Fig. 4b

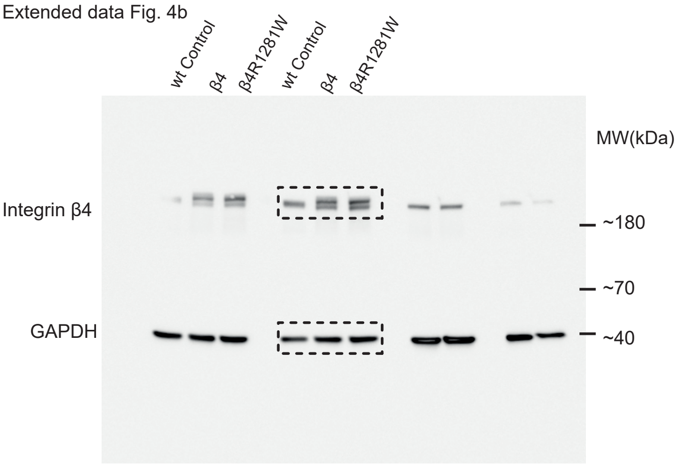

Extended data Fig. 8c

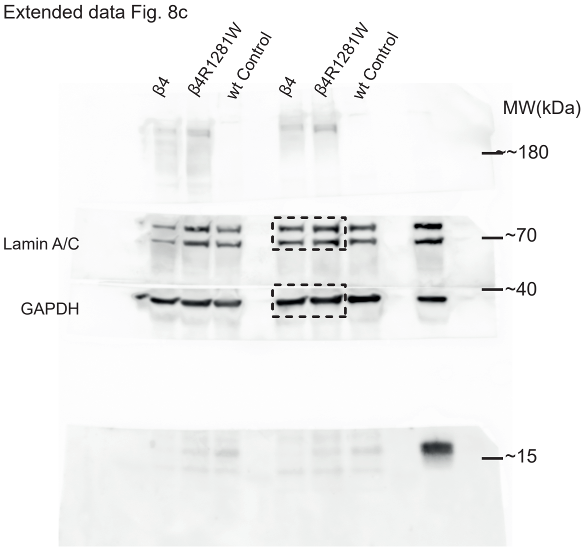

Supplement: Supplementary file 5 — Unprocessed western blots. [file 41563_2023_1657_MOESM5_ESM.pdf]
